# An EST-based analysis identifies new genes and reveals distinctive gene expression features of *Coffea arabica *and *Coffea canephora*

**DOI:** 10.1186/1471-2229-11-30

**Published:** 2011-02-08

**Authors:** Jorge MC Mondego, Ramon O Vidal, Marcelo F Carazzolle, Eric K Tokuda, Lucas P Parizzi, Gustavo GL Costa, Luiz FP Pereira, Alan C Andrade, Carlos A Colombo, Luiz GE Vieira, Gonçalo AG Pereira

**Affiliations:** 1 Centro de Recursos Genéticos Vegetais, Instituto Agronômico de Campinas, CP 28, 13001-970, Campinas-SP, Brazil; 2 Laboratório de Genômica e Expressão, Departamento de Genética, Evolução e Bioagentes, Instituto de Biologia, Universidade Estadual de Campinas, CP 6109, 13083-970, Campinas-SP, Brazil; 3 Laboratório Nacional de Biociências (LNBio), CP 6192, 13083-970, Campinas-SP, Brazil; 4 Centro Nacional de Processamento de Alto Desempenho em São Paulo, Universidade Estadual de Campinas, CP 6141, 13083-970, Campinas, SP, Brazil; 5 Embrapa Café - Instituto Agronômico do Paraná, Laboratório de Biotecnologia Vegetal, CP 481, 86001-970, Londrina-PR, Brazil; 6 Núcleo de Biotecnologia-NTBio, Embrapa Recursos Genéticos e Biotecnologia, Parque Estação Biológica, CP 02372, 70770-900, Brasília-DF, Brazil; 7 Instituto Agronômico do Paraná, Laboratório de Biotecnologia Vegetal, CP 481, CEP 86001-970, Londrina-PR, Brazil

## Abstract

**Background:**

Coffee is one of the world's most important crops; it is consumed worldwide and plays a significant role in the economy of producing countries. *Coffea arabica *and *C. canephora *are responsible for 70 and 30% of commercial production, respectively. *C. arabica *is an allotetraploid from a recent hybridization of the diploid species, *C. canephora *and *C. eugenioides*. *C. arabica *has lower genetic diversity and results in a higher quality beverage than *C. canephora*. Research initiatives have been launched to produce genomic and transcriptomic data about *Coffea *spp. as a strategy to improve breeding efficiency.

**Results:**

Assembling the expressed sequence tags (ESTs) of *C. arabica *and *C. canephora *produced by the Brazilian Coffee Genome Project and the Nestlé-Cornell Consortium revealed 32,007 clusters of *C. arabica *and 16,665 clusters of *C. canephora*. We detected different GC3 profiles between these species that are related to their genome structure and mating system. BLAST analysis revealed similarities between coffee and grape (*Vitis vinifera*) genes. Using KA/KS analysis, we identified coffee genes under purifying and positive selection. Protein domain and gene ontology analyses suggested differences between *Coffea *spp. data, mainly in relation to complex sugar synthases and nucleotide binding proteins. OrthoMCL was used to identify specific and prevalent coffee protein families when compared to five other plant species. Among the interesting families annotated are new cystatins, glycine-rich proteins and RALF-like peptides. Hierarchical clustering was used to independently group *C. arabica *and *C. canephora *expression clusters according to expression data extracted from EST libraries, resulting in the identification of differentially expressed genes. Based on these results, we emphasize gene annotation and discuss plant defenses, abiotic stress and cup quality-related functional categories.

**Conclusion:**

We present the first comprehensive genome-wide transcript profile study of *C. arabica *and *C. canephora*, which can be freely assessed by the scientific community at http://www.lge.ibi.unicamp.br/coffea. Our data reveal the presence of species-specific/prevalent genes in coffee that may help to explain particular characteristics of these two crops. The identification of differentially expressed transcripts offers a starting point for the correlation between gene expression profiles and *Coffea *spp. developmental traits, providing valuable insights for coffee breeding and biotechnology, especially concerning sugar metabolism and stress tolerance.

## Background

Coffee is the most important agricultural commodity in the world and is responsible for nearly half of the total exports of tropical products [1]. Indeed, coffee is an important source of income for many developing tropical countries. Brazil, Vietnam and Colombia account for > 50% of global coffee-production. In addition, coffee is also important to many non-tropical countries that are highly involved in coffee industrialization and commerce and are intensive consumers of coffee beverages.

Two species of the genus *Coffea *are responsible for almost all coffee bean production: *C. arabica *and *C. canephora *(approximately 70 and 30% of worldwide production, respectively). *C. arabica *is an autogamous allotetraploid (amphidiploid; 2n = 4× = 44) species originating from a relatively recent cross (≅1 mya) between *C. canephora *(or a canephoroide-related species) and *C. eugenioides*, which occurred in the plateaus of Central Ethiopia [2,3]. As a consequence of its autogamy and evolutionary history, "Arabica" coffee plants have a narrow genetic basis. This problem is amplified in the main cultivated genotypes (i.e., Mundo Novo, Catuai and Caturra), which were selected from only two base populations: Typica and Bourbon [4]. Conversely, *C. canephora *is a diploid (2n = 2× = 22), allogamous and more polymorphic *Coffea *species. In contrast to *C. arabica*, which is grown in highland environments, *C. canephora *is better adapted to warm and humid equatorial lowlands. *C. arabica *is regarded as having a better cup quality, which seems to depend on the quality and amount of compounds stored in the seed endosperm during bean maturation [5-7]. Conversely, *C. canephora *is considered more resistant to diseases and pests and has a higher caffeine content than *C. arabica *[8]. Other important differences are related to fruit maturation. Though *C. canephora *blossoms earlier, its fruit maturation is delayed in comparison to *C. arabica *[9]. Improvements in the agronomic characteristics of coffee (e.g., cup quality, pathogen and insect resistance and drought stress tolerance) are long-sought by the coffee farming-community. However, the introduction of a new trait into an elite coffee variety via conventional breeding techniques is a lengthy process due to the narrow genetic basis of *C. arabica *[4,10] and the long seed-to-seed generation cycle.

Expressed sequence tags (ESTs) provide a source for the discovery of new genes and for comparative analyses between organisms. Many EST sequencing efforts have successfully provided insights into crop plants development [11-18]. EST sequencing allows quantitative expression analyses by correlating EST frequency with the desirable traits of plant species. It also constitutes an interesting tool for the detection of tissue/stress specific promoters and genetic variation that may account for specific characteristics. Furthermore, EST analyses can provide targets for transgenesis, an interesting tool for genetic improvement of such a long generation time crop as coffee. In fact, data in coffee genetic transformation indicate the potential of this approach in molecular breeding [19,20].

Research on coffee genomics and transcriptomics has gained increasing attention recently. A Brazilian consortium (Brazilian Coffee Genome Project; BCGP) [21] was developed to investigate coffee traits by sequencing cDNA derived from a series of tissues of *C. arabica*, *C. canephora *and *C. racemosa*, a coffee species used in breeding programs for the introgression of resistance against coffee leaf miner. Concomitantly, an initiative from the Nestlé Research Center and the Department of Plant Biology at Cornell University sequenced ESTs from *C. canephora *farm-grown in east Java, Indonesia. This research group compared the EST repertoires of *C. canephora*, *Solanum lycopersicum *(tomato) and *Arabidopsis thaliana *[22,23]. Based on their analysis, it was verified that *C. canephora *and tomato have a similar assembly of genes, which is in agreement with their similar genome size, chromosome karyotype, and chromosome architecture [22]. In addition, an important platform for functional genomics that can be applied to coffee was carried out by the SOL Genomics Network (SGN; http://sgn.cornell.edu), a genomics information resource for the Solanaceae family and related families in the Asterid clade, such as *Coffea spp*. and other Rubiaceae species [23].

The availability of EST data from both of the commercially most important *Coffea *spp. prompted us to perform a wide bioinformatics analysis. In this report, we surveyed the coffee transcriptome by analyzing ESTs from *C. arabica *and *C. canephora*. Resources developed in this project provide genetic and genomic tools for *Coffea *spp. evolution studies and for comparative analyses between *C. arabica *and *C. canephora*, regarding gene families' expansion and gene ontology. We also identified *Coffea*-specific/prominent gene families using automatic orthology analysis. Additionally, we describe the annotation of differentially expressed genes according to *in silico *analysis of EST frequencies.

## Results and Discussion

### Overall *Coffea *spp. EST libraries data

**Figure 1:**
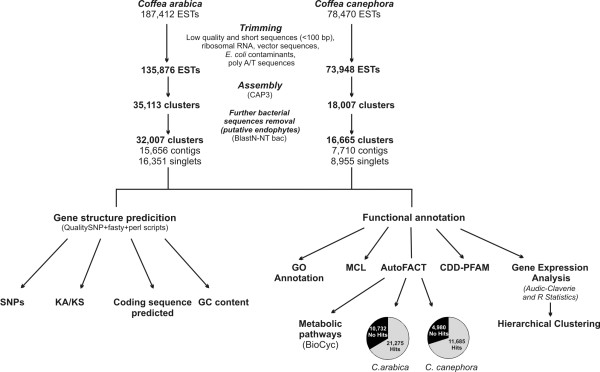
**Flow diagram of bioinformatics procedures applied in *C. arabica *and *C. canephora *transcriptomic analyses**.

To evaluate ESTs from *Coffea *spp. we collected 187,412 ESTs derived from 43 cDNA libraries produced by the Brazilian Coffee Genome Project initiative [21]. The *C. arabica *libraries represent diverse organs, plant developmental stages and stress treatments from Mundo Novo and Catuaí cultivars, excluding germinating seeds (cv Rubi) (Additional File 1). In the case of *C. canephora*, 62,823 ESTs from six cDNA libraries of the Nestlé and Cornell *C. canephora *sequencing initiative [22] and 15,647 *C. canephora *ESTs from three cDNA libraries constructed by the Brazilian Coffee Genome Project initiative [21] were collected yielding a total of 78,470 ESTs (Additional File 1). All ESTs were produced by the Sanger method, and cDNA clones were subjected only to 5' sequencing. The pipeline of *C. arabica *and *C. canephora *EST analysis is described in Figure 1.

**Figure 2:**
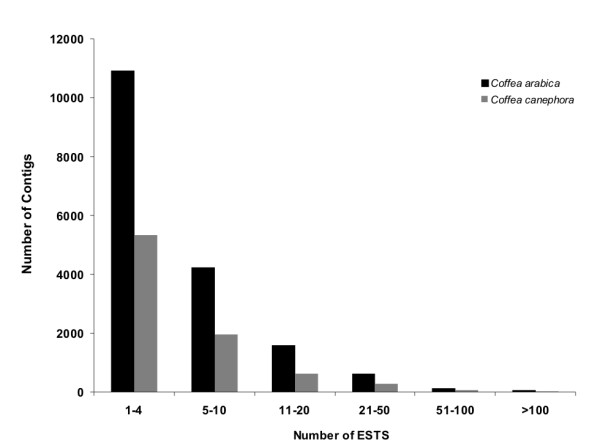
**Distribution of the number of ESTs in contigs of *C. arabica *and *C. canephora *after the assembly process**.

After trimming (i.e., vector, ribosomal, short, low quality and *E. coli *contaminant sequences removal), 135,876 *C. arabica *ESTs were assembled into 17,443 contigs and 17,710 singlets (35,113 clusters; Figure 1), and the *C. canephora *ESTs were assembled into 8,275 contigs and 9,732 singlets (18,007 clusters; Figure 1). After manual annotation, we detected some clusters similar to bacterial sequences that were not identified during trimming. Clusters were then evaluated using BLASTN against a version of NT-bac and BLASTX against the NR database. Sequences similar to bacteria were removed from further analyses. These sequences are likely derived from endophytes of coffee plants. After their removal from the dataset, the final number of clusters was 32,007 (15,656 contigs and 16,351 singlets) from *C. arabica *and 16,665 (7,710 contigs and 8,955 singlets) from *C. canephora *(Table 1). The average length of *C. canephora *and *C. arabica *clusters in the dataset was 662 bp (ranging from 100 to 3,584 bp) and 663 bp (ranging from 100 to 2,988 bp), respectively (Table 1). The number of ESTs in the *C. canephora *and *C. arabica *contigs ranged from 2 to 1,395 and 2 to 493, respectively (Figure 2). In both cases, approximately 63% were composed of ≤ 20 ESTs, and 98% of the contigs contained < 50 ESTs. We also verified the distribution of ESTs in contigs across multiple libraries. Nineteen percent of *C. arabica *contigs and 4% of *C. canephora *contigs were found in only one library (Additional File 2). The majority of *C. arabica *contigs (32%) have only two ESTs, each one from a different EST library. Due to the limited depth of sequencing and the variety of tissue samples used to construct the *C. arabica *libraries, a smoother distribution of contigs *per *library was observed in comparison with *C. canephora *(Additional File 2).

**Table 1:** Summary of *Coffea *spp. cluster datasets.

	Contigs	Average contig length	Singlets	Average singlet length	Clusters	Average cluster length
*C. arabica*	15,656	868 bp	16,351	459 bp	32,007	662 bp (ranging from 100 to 3,584 bp)

*C. canephora*	7,710	832 bp	8,955	494 bp	16,665	663 bp (ranging from 100 to 2,988 bp)

### Evaluation of GC content, SNPs and sequence similarity with other species

We evaluated the structure of *Coffea *contigs to identify the percentage of coding sequences (CDS) in our dataset using the QualitySNP program tools [24]. The mode and median length of CDS and 5' and 3' UTRs were similar to both species (Table 2). We also inspected the amount of full length CDS in our dataset, resulting in 1,189 contigs in *C. arabica *(8%) and 518 contigs in *C. canephora *(7%; Table 2).

**Table 2:** Evaluation of CDS, 5'UTR and 3'UTR of *Coffea *spp.

	Full length CDS sequences	5'UTR length (median)	CDS length (median)	CDS length (mode)	3'UTR length (median)
*C. arabica*	1,189	160 bp	836 bp	479 bp	240 bp

*C. canephora*	518	134 bp	708.5 bp	476 bp	229.5 bp

**Figure 3:**
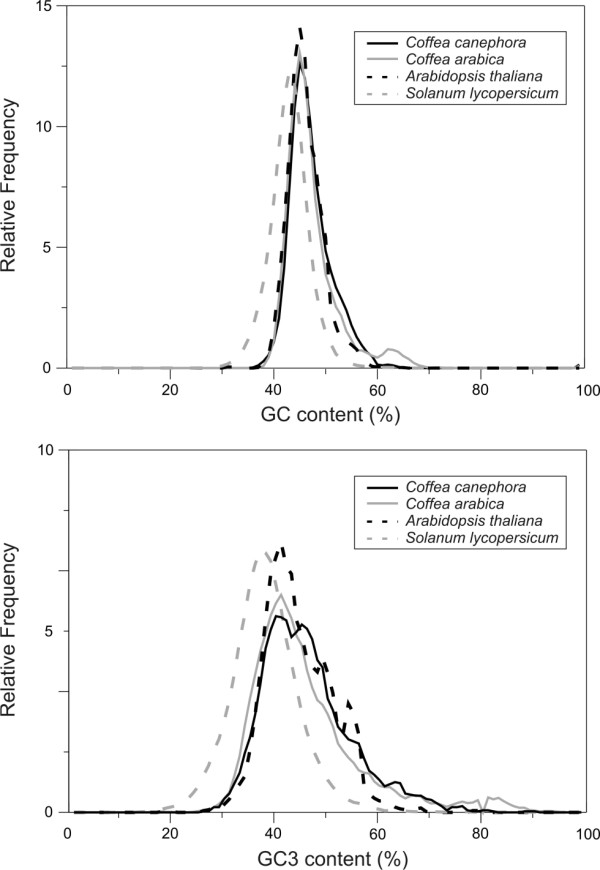
**Distribution of GC in the coding regions of *Arabidopsis thaliana*, *Solanum lycopersicum*, *C. arabica *and *C. canephora***.

Based on the annotation of CDS, we evaluated the GC content in coding regions. In general, the GC and GC3 profiles (i.e., the GC level at the third codon position) of *C. canephora *and *C. arabica *are similar to Arabidopsis and tomato. The unimodal GC distribution is a common feature of dicotyledons (Figure 3), whereas bimodal distribution is common in monocotyledons [17,25]. Nevertheless, *Coffea *spp.and Arabidopsis have a slightly higher proportion of genes with high GC content than tomato and have a more accentuated peak shift in GC3 content (Figure 3). This difference between Arabidopsis and tomato was found previously [25] and was attributed to differences in the gene samples, such as the presence of intron-retained transcripts (differentially spliced transcripts) in tomato. A more detailed inspection revealed that *C. arabica *has only one GC3 peak, while *C. canephora *has two close peaks: the first similar to that found for *C. arabica *and the other positioned toward the "GC-rich content area". This *C. canephora *pattern may be related to its outcrossing mating system because allogamous species tend to accumulate more polymorphism in the third codon position and to be more GC-rich than autogamous species [26], as is the case of Arabica coffee, tomato and Arabidopsis.

We also used QualitySNP to calculate SNPs present in *C. arabica *and *C. canephora *contigs. In the case of *C. arabica*, we selected contigs containing at least four reads, which in theory provide two copies for each allele, yielding 8,514 *C. arabica *and 3,832 *C. canephora *contigs. Approximately 53% (4,535) of the *C. arabica *contigs and 52% (2,000) of the *C. canephora *contigs were found to contain SNPs (Additional File 3). Similar to other reports [27-29], more transitions than transversions were found for both species (Additional File 3), likely reflecting the high frequency of cytosine to thymine mutation after methylation. The frequency of SNPs in *C. arabica *was 0.35 SNP/100 bp, almost double the *C. canephora *SNP frequency (0.19 SNP/100 bp). Similarly, Lashermes et al. [3] and Vidal et al. [30] indicated that Arabica has a level of internal genetic variability almost twice that present in *C. canephora*. The majority of polymorphisms found in both species was bi-allelic (99.8% for *C. arabica *and 99.5% for *C. canephora*), with a low percentage of tri-allelic and no tetra-allelic SNPs (Additional File 3)

We next used AutoFACT [31] to evaluate the putative functions of the two *Coffea *datasets. The results of BLASTX against the non-redundant protein sequence database (NR; E-value cutoff of 1e^-10^) available at AutoFACT were inspected to evaluate the similarity of *Coffea *clusters with proteins deposited in GenBank. Approximately 68% of *C. arabica *and 71% of *C. canephora *clusters have significant sequence similarity (E-value ≤ 1e^-10^) with genes in the databank. The remaining clusters represented sequences with lower E-value scores (E-value > 1e^-10^) designated as "no-hits" (Table 3). Because *C. arabica *and *C. canephora *are species from the Rubiaceae family, which have few sequences deposited in the NR database, we expected that sequences from other species in the Asteridae clade (e.g., members of the Solanaceae family *S. lycopersicum, S. tuberosum *and *Nicotiana tabacum*) would be the most similar to *Coffea *sequences. However, the majority of *Coffea *clusters have higher similarity with *Vitis vinifera *sequences (~40%), a species from the Rosids clade, followed by the other rosids Arabidopsis (~5.5%) and *Populus trichocarpa *(~3.5%). The top hits of Coffee sequences with Solanaceae range from 1 to 2% (Table 3). We then compared the *Coffea *sequences with a database containing contigs from the plant EST databank TIGR, the plant transcript database http://plantta.jcvi.org and GeneIndex Plants http://compbio.dfci.harvard.edu/tgi/plant.html, which have a higher amount of Solanaceae data. For both *C. arabica *and *C. canephora*, *N. tabacum *was the species with more top hits (11.15 and 11.59%, respectively), followed by *V. vinifera *(10.34 and 10.03%), *S. lycopersicum *(6.5 and 5%) and *S. tuberosum *(5 and 4.8%; data not shown). We believe that the most parsimonious hypothesis for these results is related to phylogenetic issues. Grape is basal to the rosids clade and did not undergo whole genome duplication (WGD) events, such as Arabidopsis, thus being theoretically more similar to the rosids paleohexaploid ancestor [32,33]. Analysis of genomic sequences from the asterid common monkey flower (*Mimulus guttatus*) revealed extensive synteny with grape, suggesting that paleohexaploidy antedates the divergence of the rosid and asterid clades [33]. Notably, recent data prove that there is a high level of collinearity between diploid *Coffea *and *V. vinifera *genomic regions [34], and that these species derive from the same paleohexaploid ancestral genome [35]. Intensive genomic analyses are currently underway to more deeply compare the genomes of rosids and asterids species.

**Table 3:** Predicted *C. arabica *and *C. canephora *gene comparisons.

*Coffea arabica*		
**Species**	# Hits*	**% Hits**

*Vitis vinifera*	13,855	43.29%
*Arabidopsis thaliana*	1,846	5.77%
*Populus trichocarpa*	1,161	3.63%
*Oryza sativa*	643	2.01%
*Nicotiana tabacum*	641	2.00%
*Solanum tuberosum*	428	1.34%
*Solanum lycopersicum*	392	1.22%
*Medicago truncatula*	149	0.47%
*Catharanthus roseus*	115	0.36%
*Glycine max*	104	0.32%
Others	1,941	6.06%
No hits	10,732	31.66%

***Coffea canephora***		

**Species**	**# Hits**	**% Hits**

*Vitis vinifera*	7,427	44.57%
*Arabidopsis thaliana*	972	5.83%
*Populus trichocarpa*	639	3.83%
*Oryza sativa*	372	2.23%
*Nicotiana tabacum*	362	2.17%
*Solanum tuberosum*	232	1.39%
*Solanum lycopersicum*	225	1.35%
*Medicago truncatula*	105	0.63%
*Solanum demissum*	64	0.37%
*Catharanthus roseus*	56	0.32%
Others	1,231	7.39%
No hits	4,980	29.88%

* Each coffee cluster was compared to all of the proteins from the organisms listed. The BLASTX score was defined as 1e^-10^.

To gain insight into the molecular evolution of protein coding genes in the two *Coffea *species analyzed, we estimated the rates of synonymous (KS, silent mutation) and non-synonymous (KA, amino-acid altering mutation) substitutions generated by QualitySNP analysis, and performed the KA/KS test for positive selection of each hypothetical gene. KA/KS is a good indicator of selective pressure at the sequence level. Theoretically, a KA/KS >1 indicates that the rate of evolution is higher than the neutral rate. Conversely, a gene with KA/KS < 1 has a rate of evolution less than the neutral rate [36]. As in other plant species [37,38], most genes in *C. arabica *and *C. canephora *appear to be under purifying selection (KA/KS < 1), indicating that the majority of protein-coding genes are conserved over time as a result of selection against deleterious variants.

The correlation between AutoFACT annotations with KA/KS analysis allowed the detection of genes with low KA/KS ratios, such as those encoding proteins involved in photosynthesis, morphogenetic development and translation (Additional File 4). The majority of these proteins have been shown to be highly conserved and to suffer strong purifying selection [37]. Analyzing the genes with the highest KA/KS, we identified effector proteins and transcription factors related to biotic and abiotic stress and proteins involved in oxidative respiration (Additional File 4). These results are in accordance with previous reports, which show that genes acting in response to stress are often positively selected for diversification due to the competition with the evolving effector proteins of pathogens [37,39].

### Metabolic Pathways

We constructed hypothetical metabolic maps for both *C. arabica *and *C. canephora *using BioCyc [40]. After manual annotation, 345 pathways in *C. arabica *and 300 pathways in *C. canephora *were detected. *C. arabica *pathways included 3,366 enzymes in 1,807 enzymatic reactions. In the case of *C. canephora*, 1,889 enzymes were present in 1,653 enzymatic reactions. The almost two-fold difference in the number of enzymes between the two coffee species is related to the number of ESTs annotated for each species. Therefore, assigning the presence/absence of a pathway in one *Coffea *species relative to the other should be done carefully. Further, the number of *C. arabica *enzymatic reactions may be underestimated due to duplicated genes in *C. arabica*, each one most likely derived from a different ancestor (*C. canephora *and *C. eugenioides*), because that two enzymatic reactions in *C. arabica *may be annotated as only one. The data for the fully annotated pathways are available at the website http://www.lge.ibi.unicamp.br/coffea.

### Protein Domains

**Figure 4:**
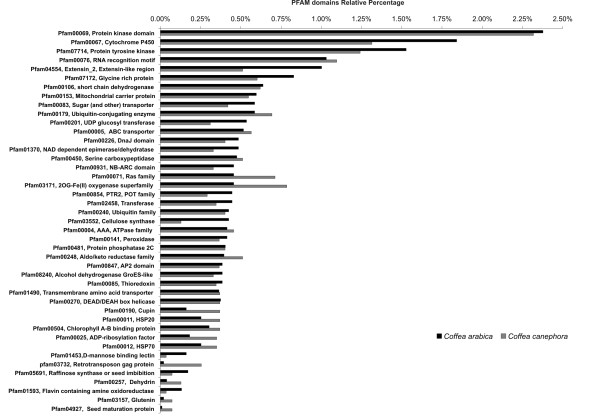
**Comparative chart between the relative percentage of Pfam domains in *C. arabica *and *C. canephora *EST databases**.

We performed a comparison of *C. arabica *and *C. canephora *gene clusters with the CDD-PFAM databank to catalog the protein domains present in the *Coffea *EST datasets. The submission of the clusters to RPS-BLAST resulted in 30% (9,886) of *C. arabica *and 32% (5,478) of *C. canephora *clusters containing an assigned domain. To compare the prevalence of protein domains in *Coffea *species, the number of clusters assigned to each domain was normalized by dividing by the total number of clusters containing a domain. Serine threonine kinases (Pfam00069), cytochrome P450 monooxygenases (Pfam00067), tyrosine kinases (Pfam07714) and proteins containing RNA recognition motifs (RRM; Pfam00076) are among the top 20 PFAM families in *Coffea *species (Additional File 5). Next, we plotted the percentage of protein domains in *Coffea *datasets in a comparative histogram. Protein domain analysis revealed significant differences between the two species datasets (Figure 4). For example, *C. arabica *contains more cytochrome P450 monooxygenases, tyrosine kinases, extensin-like proteins, glycine-rich proteins, sugar transporters, UDP glucosyl- transferases, NAD-dependent epimerases, DNA-J proteins, NB-ARC proteins, cellulose synthases, raffinose synthases, D-mannose-binding lectins and flavin amine oxidoreductases than *C. canephora *(Figure 4). In contrast, the *C. canephora *dataset contains a higher percentage of transcripts coding for proteins containing RRM motifs, ubiquitin conjugation enzymes, ABC transporters, Ras/Rab/Rac proteins, 2-OG oxygenases, cupin proteins, HSP20 s, HSP70 s, ADP-ribosylation factors, dehydrins, glutenins and seed maturation proteins (Figure 4). Despite these dissimilarities between datasets may be caused by the different tissues used for constructing the *C. arabica *and *C. canephora *cDNA libraries, such results offer clues for further comparative research.

One noteworthy difference between domains is the greater percentage of proteins containing the retrotransposon gag protein domain (Pfam03732) in *C. canephora *(0.26%) than in *C. arabica *(0.02%). This domain is found in LTR-retrotransposons, the most widespread transposable element (TE) family in plants [41]. Lopes et al. [42] found that *Coffea *species harbor fewer TE-cassettes (> 0.04%) than would be expected from the translation of TE-containing transcripts (0.23%). These authors hypothesized that such incongruence may either be a consequence of the exonization/exaptation of TE fragments or an indication of the tolerance of alternatively spliced "TE-invaded" mRNAs that do not encode functional proteins. A more detailed investigation is in progress to explore the diversity and differences between *Coffea *spp. TEs (F.R. Lopes, M.F. Carazzolle, G.A.G. Pereira, C.A. Colombo, C.M.A. Carareto; unpublished data).

### Gene Ontology Analysis and Annotation

**Figure 5:**
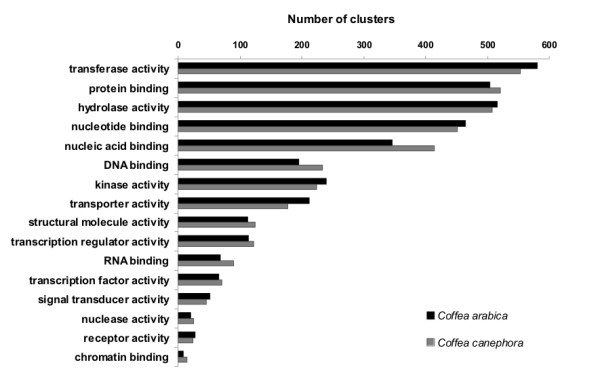
**Distribution of *C. arabica *and *C. canephora *clusters with putative functions assigned through annotation using molecular function gene ontology**.

A functional annotation was performed by mapping contigs assembling onto gene ontology (GO) structures [43]. Approximately 38% of *C. arabica *and 49% of *C. canephora *clusters were mapped with a biological process, and 43 and 55% were mapped with a molecular function. These differences reflect the greater amount of *C. arabica *ESTs in the libraries compared to *C. canephora *and are likely related to the fact that some tissues used in *C. arabica *libraries (i.e., callus) were not extensively studied, resulting in genes with unassigned ontologies. To compare the gene ontologies, the amount of sequences associated with each term was normalized (see methods), and then hypergeometric statistics were applied [44]. To compare GO data with our other protein-related analysis, we focused our evaluation on molecular activity ontology. We observed that *C. arabica *has a greater amount of transcripts coding for proteins with catalytic activity, transferase activity and transporter activity than *C. canephora *(Figure 5). In accordance, the CDD-PFAM analyses showed that *C. arabica *had a greater percentage of cellulose synthases, raffinose synthases, UDP-glucuronosyl transferases, secondary metabolism-related transferases, ABC transporters and sugar transporters (Figure 4; Additional File 5). The evidence that transcripts coding for proteins related to sugar metabolism and transport are more prevalent in *C. arabica *than in *C. canephora *may be related to the high content of sugars (especially sucrose) in fruits of Arabica plants, one of the traits that provides a better cup quality (see below). In contrast to *C. arabica*, *C. canephora *has more proteins annotated as containing binding activity, which is extended for the binding activity branch child terms of nucleic acid binding, DNA and RNA binding activities, transcription regulation and transcription factor activities (Figure 5). These data are also in agreement with our domain analysis (Figure 4; Additional File 5), indicating a higher percentage of Ras/Rac/Rab GTPase proteins, including regulators of vesicle biogenesis in intracellular traffic, ADP-ribosylation factors and proteins containing RRM and G-patch motifs, involved in RNA binding activity [45].

### Orthologous Family Clustering: Searching for Coffee-Specific Families

To identify proteins that are hypothetically specific or at least prominent in *Coffea *spp. in comparison to other species, we applied OrthoMCL, a graph-clustering algorithm designed to identify homologous proteins based on sequence similarity [46,47]. Two different types of datasets were used in this analysis: i) the annotated proteins from the available complete genomes of *A. thaliana*, *V. vinifera*, *Oryza sativa*, *Ricinus communis *and *Glycine max *and ii) the proteins predicted by FrameDP software [48] from the available ESTs assemblies for *C. arabica*, *C. canephora *and *S. lycopersicum*. Based on the fact that some genes are not picked in EST libraries, the evaluation of *Coffea *spp. gene family retraction was not performed (i.e., the absence of a gene does not mean that it is not present in the genome but rather that it is expressed in a minor amount).

We identified 24,577 different families using the eight aforementioned species. The majority of families were ubiquitous, being present in all analyzed species. The top three OrthoMCL families in *Coffea *spp. are: i) a family composed of serine/threonine kinases (family 1), ii) pentatricopeptide repeat-containing proteins (family 2) and iii) cytochrome P450 monooxygenases (family 6; Table 4). The analysis was focused on the annotation of families that appeared to be specific from *Coffea *species or that are prominent in those EST datasets. In *C. arabica*, we highlight family 544, which contains proteins similar to the cysteine proteinase inhibitors cystatins. This family includes 21 members in *C. arabica*, six in *C. canephora *and only one member in the grape genome (Table 4). Two other proteins families composed of cystatin-like proteins (families 2703 and 11594) are also prominent in coffee plants. Other protein families that appear to be prominent/specific in *C. arabica *include small secreted glycine-rich proteins similar to *Panax ginseng *[49] (families 1231, 4031 and 11588), NBS-LRR resistance proteins (families 453, 3289 and 2722), Pin2-like serine proteinase inhibitors (families 7241 and 10273), conserved proteins of unknown function (families 10956, 11617, 12384, 12386, 11626 and 13353), proteins not previously described (no hits; families 14110 and 14413), etc. (Table 4). In *C. canephora*, the "species-specific/prominent" gene families include those encoding miraculin-like proteins (family 14813), *C. canephora*-specific invertase inhibitors (family 14814), small secreted glycine-rich proteins (family 11055), Ty3 Gypsy-like retrotransposons (family 10952), kelch repeat phosphatases (family 14392), 2 S albumin storage proteins (family 14392), etc. (Table 4). Five families are specific or prominent in both *C. arabica *and *C. canephora *when compared to the other species analyzed. Two of these contain proteins not previously described (no hits, families 10281 and 12375). The other three include proteins similar to rapid alkalinization factor (RALF, family 8498), GTP binding proteins (family 9023) and proline-rich extensins (family 12371; Table 4).

**Table 4:** OrthoMCL analysis of *C. arabica *and *C. canephora*, highlighting prominent and specific families in *Coffea *spp

OrthoMCL family ID	*Coffea arabica*	*Coffea canephora*	*Vitis Vinifera*	*Solanum lycopersicum*	*Glycine max*	*Ricinus communis*	*Oryza sativa*	*Arabidopsis thaliana*	Manual Annotation*
1	446	189	1402	808	2532	1378	813	847	Serine-threonine kinase

2	152	51	580	212	967	461	478	447	PPR repeat protein

6	84	41	193	123	226	99	101	108	Cytochrome P450

544	21	6	1	-	-	-	-	-	Cystatin

453	14	4	1	7	3	1	1	1	NBS LRR resistance protein

1231	13	5	-	-	-	-	-	-	Small secreted glycine-rich protein

4031	10	-	-	-	-	-	-	-	Glycine-rich protein

1510	7	1	1	-	2	1	1	3	UDP-glucosyltransferase

2703	6	3	-	1	1	-	1	-	Cysteine proteinase inhibitor like protein

3289	6	-	1	-	2	-	2	-	NBS LRR resistance protein

5056	6	1	-	1	-	-	-	-	Alcohol dehydrogenase

2306	5	1	-	2	1	1	2	-	Cytochrome P450

2722	5	1	-	1	1	2	1	1	NBS LRR resistance protein

3294	5	-	1	-	3	-	1	1	Poly-A binding protein

3303	5	1	2	1	-	-	-	1	NADPH-dependent cinnamyl alcohol dehydrogenase

3305	5	2	1	2	-	-	-	-	Specific tissue protein 2

4049	5	2	1	1	-	-	1	-	Sugar transport protein

4070	5	-	1	1	3	-	-	-	Cytochrome P450

7241	5	1	1	-	-	-	1	-	Potato type II serine proteinase inhibitor family

10956	5	-	-	-	-	-	-	-	Hypothetical protein

7610	4	1	-	1	-	-	-	1	Ubiquitin-conjugating enzyme

7611	4	1	-	1	1	-	-	-	P-glycoprotein ABC

7613	4	-	-	2	1	-	-	-	Hexose transporter

9014	4	1	-	-	-	1	-	-	GH3 family protein/Indole-3-acetic acid-amido synthetase

10273	4	1	-	-	-	-	-	-	Potato type II serine proteinase inhibitor family

11588	4	-	-	-	-	-	-	-	Small secreted glycine-rich protein

11617	4	-	-	-	-	-	-	-	Hypothetical protein

12384	4	-	-	-	-	-	-	-	Hypothetical protein

12385	4	-	-	-	-	-	-	-	Defensin/gamma thionin

12386	4	-	-	-	-	-	-	-	Hypothetical protein

7324	3	2	-	-	2	-	-	-	Helix-loop-helix DNA-binding protein

9019	3	-	-	1	-	1	-	-	Zinc/iron transporter

9830	3	-	3	-	-	-	-	-	Eukaryotic initiation factor (eIF1)/SU1

10271	3	1	-	-	-	-	1	-	Metallothionein

10276	3	-	-	-	-	1	-	1	SEC14 cytosolic factor family protein

10293	3	-	-	1	1	-	-	-	ABC transporter

10300	3	1	-	-	1	-	-	-	Phytochrome B/histidine kinase

10309	3	1	-	1	-	-	-	-	Oxidoreductase

11058	3	-	1	1	-	-	-	-	ATP-binding cassette transporter

11594	3	-	-	-	-	-	-	1	*A. thaliana*-related cystatin

11600	3	-	-	-	-	-	1	-	Alcohol dehydrogenase

11607	3	1	-	-	-	-	-	-	CAAX amino-terminal protease

11626	3	1	-	-	-	-	-	-	Hypothetical protein

13353	3	-	-	-	-	-	-	-	Hypothetical protein

13392	3	-	-	-	-	-	-	-	GDP-D-mannose 4,6-dehydratase

14410	3	-	-	-	-	-	-	-	No hits found

14413	3	-	-	-	-	-	-	-	No hits found

14414	3	-	-	-	-	-	-	-	Aspartate aminotransferase superfamily protein

14418	3	-	-	-	-	-	-	-	HAT transposase element

14420	3	-	-	-	-	-	-	-	Protein translation factor SUI1

8498	2	5	-	-	-	-	-		Rapid Alkalinization Factor (RALF)-like protein

9023	2	3	-	-	-	1	-	-	GTP binding protein

10281	2	3	-	-	-	-	-	-	No hits found

12371	2	2	-	-	-	-	-		Hydroxyproline-rich glycoprotein/extension

12375	2	2	-		-	-	-	-	No hits found

1715	-	4	1	2	1	8	-	-	Viroid polyprotein ORF4 protein

6375	-	4	2	1	1	-	-	-	NBS LRR resistance protein

9679	-	3	1	-	1	1	-	-	Replication factor A 1

10952	-	3	1	-	-	1	-	-	LTR retrotransposon

11055	-	5	-	-	-	-	-	-	Small glycine-rich protein

14392	-	3	-	-	-	-	-	-	Kelch repeat-containing phosphatase

14397	-	3	-	-	-	-	-	-	Albumin/sulfur-rich seed storage protein

14809	-	3	-	-	-	-	-	-	Hypothetical protein

14813	-	3	-	-	-	-	-	-	Miraculin-like protein

14814	-	3	-	-	-	-	-	-	Invertase inhibitor

* Annotation based on BLASTX-NR (E-value 1e^-5^).

### *In silico *Evaluation of Gene Expression in *C. arabica *and *C. canephora*

We correlated the AutoFACT annotation results with the distribution of contigs in the *C. arabica *and *C. canephora *libraries (Additional Files 6 and 7). The majority of the most widely distributed genes is related to RNA processing, translation, protein turnover and protein folding. This was an expected result because these biological processes are ubiquitous and indispensable for cellular homeostasis (Additional File 6). In Arabica, the most widely expressed contigs encode a papain-like cysteine (cys) proteinase (234 ESTs) and a polyubiquitin (207 ESTs), each one distributed among 30 libraries, followed by glyceraldehyde 3-phosphate dehydrogenase (*GAPDH*; 162 ESTs) and a heme-containing peroxidase (245 ESTs), both distributed among 29 libraries (Additional File 6). Both polyubiquitin and *GAPDH *were previously tested as suitable reference genes for qPCR expression analysis in *C. Arabica *[50-52], which reinforces the accuracy of our bioinformatics analyses. The data presented here provide additional genes to be tested for normalization of qPCR, an essential procedure to avoid misinterpretation when measuring gene expression [53]. The lack of libraries from diverse tissues does not allow reliable inferences about the ubiquity of genes in *C. canephora*. However, the most widely expressed contig (22 ESTs in nine libraries) encodes a putative VTC2 protein, a GDP-D-glucose phosphorylase involved in ascorbic acid biosynthesis [54], suggesting the synthesis of ascorbate throughout fruit development in *C. canephora*, which is likely used as an antioxidant and as a cofactor for dioxygenases.

The evaluation of the contigs distribution in *Coffea *libraries also revealed the contigs containing the most redundant (most highly expressed) ESTs (Additional File 7). In *C. arabica*, a contig encoding a RuBisCo small subunit was found to be the most highly expressed gene, followed by a contig encoding a putative class III chitinase (Additional File 7). Among the top 20 most expressed ESTs are genes involved in detoxification and reactive oxygen species (ROS) tolerance and genes related to biotic and abiotic stress. These annotations may be biased by the significant amount of ESTs derived from biotic or abiotic stressed tissues (Additional File 1). Two genes encoding seed storage proteins (2 S albumin and 11 S globulin) were the most highly expressed genes in the *C. canephora *dataset, a result similar to that described by Lin et al. [22] (Additional File 7). The use of regulatory elements of these highly expressed genes may be an excellent tool for conferring strong expression to a target gene in transgenesis approaches.

**Figure 6:**
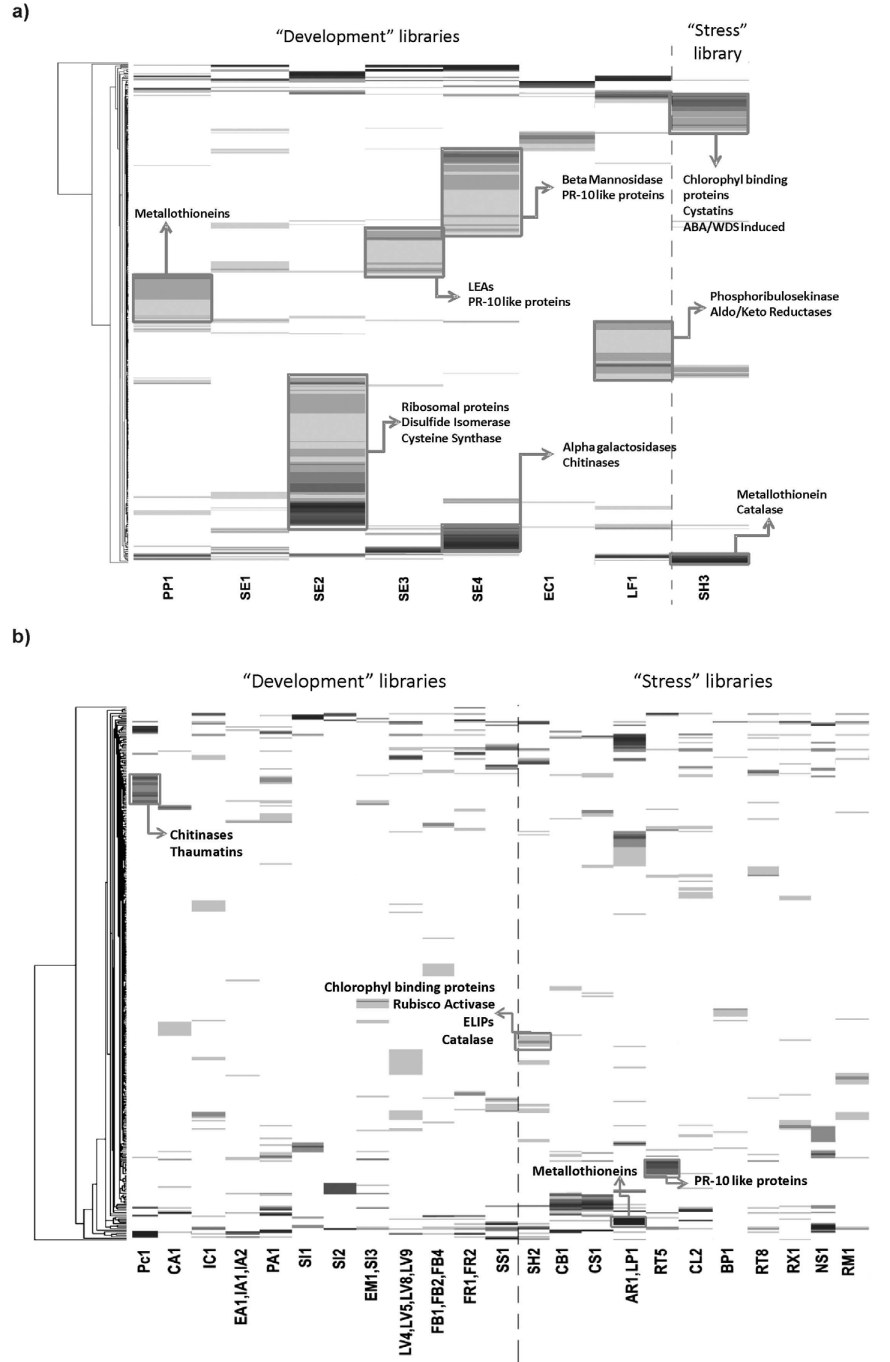
**Hierarchical clustering of coffee cDNA libraries and clusters based on EST distribution**. a) *C. canephora *hierarchical clustering of 443 clusters differentially expressed vs. the eight cDNA library assemblies. b) *C. arabica *hierarchical clustering of 331 clusters differentially expressed vs. the 23 cDNA library assemblies. Hierarchical clustering was performed using a correlation matrix constructed from EST frequencies for differentially expressed *C. arabica *and *C. canephora *contigs. Black intensity designates relative transcript abundance in a given library, as inferred from EST frequency within each contig. Library abbreviations correspond to the following descriptions: *C. canephora*: LF; young leaves, PP1; pericarp, all developmental stages; SE1; whole cherries,18 and 22 weeks after pollination; SE2, whole cherries,18 and 22 weeks after pollination; SE3: endosperm and perisperm, 30 weeks after pollination SE4; endosperm and perisperm, 42 and 46 weeks after pollination; EC1: embriogenic calli; SH1: leaves from water deficit stressed plants; and SH3: leaves from water deficit stressed plants (drought resistant clone). *C. arabica*: PC1, *C. arabica *non-embryogenic cell line induced with 2,4-D; CA1, non-embryogenic calli; IC1, *C. arabica *non-embryogenic cell line without 2,4-D; EA; EA2, *C. arabica *embryogenic calli; IA2, *C. arabica *embryogenic cell line induced with 2,4-D; PA1, primary embryogenic *C. arabica *calli; EM1, zygotic embryo from mature germinating seeds; SI3, germinating whole seeds; LV4, young leaves from orthotropic branches; LV5, young leaves from orthotropic branches; LV8, mature leaves from plagiotropic branches; LV9, mature leaves from plagiotropic branches; FB1, floral buds at developmental stages 1 and 2; FB2, floral buds at developmental stages 1 and 2; FB4, floral buds at developmental stages 3 and 4; FR1, floral buds, pinhead fruits, fruit developmental stages 1 and 2; FR2, floral buds, pinhead fruits, fruit developmental stages 1 and 2; SS1, well-watered field plant tissues; SH2, water-stressed plant tissues; CB1, suspension cells treated with acibenzolar-S-methyl and brassinosteroids; CS1, suspension cells under osmotic stress; AR1, leaves treated with arachidonic acid; LP1, plantlets treated with arachidonic acid; RT5, roots with acibenzolar-S-methyl; CL2, hypocotyls treated with acibenzolar-S-methyl; BP1, suspension cells treated with acibenzolar-S-methyl; RT8, root suspension cells under aluminum stress; RX1, *Xyllela *spp.-infected stems; NS1, nematode-infected roots; and RM1, leaves infected with leaf miner and coffee leaf rust.

To identify genes uniquely or preferentially expressed in specific coffee EST libraries, R statistics [55] and Audic Claverie (AC) statistics [56] were used through IDEG6, a web tool for the statistical analysis of gene expression data [57]. Libraries containing < 300 ESTs were discarded from these analyses, because libraries with a small amount of ESTs tend to disturb the prediction of differentially expressed genes. After some manual clusterization, we observed that several libraries derived from the same tissues (EA1, IA1 and IA2; EM1 and SI3; LV4, LV5, LV8 and LV9; FB1 and FB4; and FR1 and FR2) present the same set of genes differentially expressed in comparison to the other libraries. Thus, they were combined for further analyses. After evaluating statistical data, the merging of AC and R statistical analyses resulted in 331 contigs from *C. arabica *and 443 contigs from *C. canephora*. Thereafter, hierarchical clustering was applied to this data using a correlation matrix constructed from EST frequencies for differentially expressed *C. arabica *and *C. canephora *contigs (Figure 6; Additional File 8). The clustering results indicated that the differences among *C. canephora *libraries were more evident than in *C. arabica*, likely due to the small number of libraries of the former (Figure 6A and 6B).

The libraries were manually separated into two groups: "development" libraries, derived from tissues that did not suffer stress; and "stress" libraries that were constructed using RNA from plants challenged with biotic or abiotic stress-triggering factors. This expression "fingerprinting" provides a guideline for the isolation of promoters that regulate expression in specific tissues or stress conditions. Brandalise et al. [58] applied a similar strategy in the isolation of a *C. arabica *promoter that drives stress-responsive expression in leaves. Some genes with agronomical importance or with interesting expression profiles depicted in Figure 6 are discussed in more details in the following section. The full annotation of differentially expressed genes can be accessed at http://www.lge.ibi.unicamp.br/coffea.

### Functional Classification of Differentially Expressed Genes and Prevalent Protein Families in *C. arabica and C. canephora*

Based on the results of protein domain annotation, GO analysis, OrthoMCL data and Expression Hierarchical Clustering, we established functional categories to elucidate putative gene expression and its consequences in coffee development and environmental adaptations.

### Genes related to plant defense

#### Pathogenesis related proteins (PR)

PRs are a heterogeneous group of plant proteins, inducible by biotic stresses [59,60]. Some of these proteins are effectors against pathogens and insects, while others are involved in reestablishing homeostasis after the stress [59].

Defensins or gamma-thionins (PR-12) are small, cationic, Cys-rich proteins structurally and functionally related to biocide defensins previously characterized in mammals and insects [61]. All EST reads that compose contigs encoding gamma-thionins from OrthoMCL family 12385 were expressed in tissues treated with benzothiadiazole - BTH (BP1, CL2) or infected with nematodes (NS1). This OrthoMCL family was *C. arabica*-specific (Table 4), perhaps due to the lack of EST libraries from *C. canephora *plants treated with BTH. However, their specificity in Arabica suggests that these proteins rapidly evolved in *Coffea *spp., acquiring specific structural traits important for *Coffea *adaptation to pathogens.

The PR-10 protein family is a large group of PR proteins that are considered allergenic and exert ribonuclease activity, which is paralleled with cytokinin binding and anti-pathogenic roles [62]. In *C. arabica*, a PR-10 was found to be highly expressed in an incompatible reaction against the causative agent of coffee leaf rust, the biotrophic fungus *Hemileia vastatrix *[63]. A PR-10 from *C. arabica *(CaContig15067) was predicted to be more expressed in suspension cells treated with aluminum (Additional File 8). Concerning *C. canephora*, we observed an expression prevalence of PR-10 genes in late stages of fruit development (SE3 e SE4; Additional File 8). A proteomic analysis indicated that a *C. arabica *PR-10 was expressed only in the endosperm but not in zygotic embryos [64]. This result is similar to that found by Botton et al. [65], who reported the accumulation of a peach PR-10 during the fruit ripening stage.

One interesting result was the presence of a relatively large amount of chitinases (four contigs) and thaumatins (six contigs) in *C. arabica *calli libraries (PC1, EA1, IA1, IA2 and PA1; Additional File 8; Figure 6B). Several reports indicate the participation of these PR proteins not only in plant defense but also during somatic embryogenesis [66-69]. The chitinases are hypothesized to have signaling functions during embryogenesis, because these proteins are able to rescue somatic embryos beyond globular stage [70]. Moreover, arabinogalactan proteins (AGPs), chitinases and thaumatins secreted in suspension-culture cells can promote the production of somatic embryos [69,71]. Our data strongly indicate a role for these PRs during coffee embryogenesis.

#### Resistance Genes

Most of the disease resistance genes (R genes) in plants encode nucleotide-binding site leucine-rich repeat (NBS-LRR) proteins. They are engaged in the recognition of pathogens, being considered specific determinants of the plant immune response [72,73]. Upon annotation of OrthoMCL gene families, we detected 91 clusters and 36 clusters of CC-NBS-LRR proteins in *C. arabica and C*. *canephora*, respectively. In addition, some CC-NBS-LRR families were prevalent in *C. arabica *(Families 453, 3289, 2722) and in *C. canephora *(Family 6375; Table 4). The majority of clusters have higher identity with the PRF protein from tomato (with the exception of CaContig16622, which is more similar to RPP8 and LOV1 proteins). In a seminal report concerning the evaluation of resistance genes in coffee, 43 resistance gene analogues (RGAs) from both *C. arabica *and *C. canephora *were isolated, and it was verified that all RGAs are from the CC-NBS-LRR subfamily [74]. Nevertheless, we identified a *C. arabica *contig analogous to TIR-NBS-LRR proteins (CaContig7327), with similarity to the nematode resistance potato proteins Gro1 [75] and Arabidopsis TAO1 protein [76]. The extensive retraction (almost disappearance) of *Coffea s*pp. TIR-NBS-LRR proteins is similar to that described in cereals and sugar beet [77,78] and likely resulted from independent gene loss events in such different plant lineages [74,77,78]. The implications of the loss of TIR-type NBS-LRR genes and diversification of CC-NBS-LRRs deserve special attention in the understanding of coffee defense mechanisms.

### Genes Related to Abiotic Stress and Detoxification

Genes related to abiotic stresses are potentially important in the recent scenario of harsh environmental changes, such as the increase of extreme temperatures and drought periods. Coffee plantations are threatened by global warming due to coffee's susceptibility to high temperatures and drought when these stresses occurs during flowering and fruit development [79]. The understanding of the relationship between tolerance/susceptibility mechanisms and abiotic stress is essential for the prospection of biotechnological and crop management strategies in coffee.

We inspected the genes that were more expressed in *C. arabica *drought stressed plants (SH2) in comparison to well-watered plants conditions (SS1). Genes encoding RuBisCo activases (CaContig 5581 and 14729), a putative photosystem II type I chlorophyll a/b-binding (CAB) protein (CaContig5621) and a PSI-E subunit of photosystem I (CaContig5564) were preferentially expressed in the SH2 library (Additional File 8; Figure 6). Cramer et al. [80] also found similar expression patterns with RuBisCo activase and CAB proteins during water and salinity stresses in grapevines. In drought stress, RuBisCo activase augments RuBisCo activity that is diminished as a consequence of a lower stomatal conductance caused by diffusion limitations through stomata and mesophyll [80]. Damages in PSII proteins are associated with the decrease of PSII chemistry caused by ROS [81]. The increase of photosystem I and II genes (CAB and PSI-E subunit) may be a mechanism to sustain photosystems susceptible to ROS attack [80]. These results indicate that the activation of the photosynthetic apparatus is a mechanism of drought stress mitigation in coffee plants.

Catalase controls H_2_O_2 _concentrations by dismuting H_2_O_2 _to water and oxygen. Montavon and Bortlik [82] detected increasing of catalase activity throughout coffee grain maturation. Among genes preferentially expressed in SH2 (Additional File 8; Figure 6A) CaContig13838 has similarity to Arabidopsis catalase 2, which is activated by drought stresses [83], supporting its involvement in the dehydration response in *C. arabica*. Another contig preferentially expressed in the SH2 library (CaContig13998) is similar to early light-induced proteins (ELIPs), thylakoid-target proteins that are similar to light harvesting complex (LHC) proteins (Additional File 8; Figure 6B). ELIPs are reported to be up-regulated during various environmental stresses, such as cold and drought, and during fruit ripening [84,85]. ABA/WDS are proteins C-terminally enriched in His and Lys and are induced during ripening in pummel [86] and under water deficit stress in loblolly pine [87]. CaContig1691 appears to be one of the most expressed in water deficit stressed plants (Additional File 8; Figure 6B).

Other genes encoding proteins related to drought stress, such as dehydrins, metallothioneins and LEAs, were not differentially expressed in the SH2 library. However, we detected interesting profiles for these genes, especially for dehydrins and LEAs during fruit maturation and for metallothioneins preferentially expressed in libraries from plants treated with arachidonic acid, a polyunsaturated fatty acid present in pathogens (further details in Additional File 9).

### Plant Hormones: Auxin Regulation Genes and RALF-like Peptides

Plant hormones (phytohormones) are crucial for a series of developmental mechanisms, such as organ initiation and development, resistance to stress and reproduction. Auxins are the most studied class of phytohormones, being implicated in cell division, cell elongation and cell differentiation [88]. Using OrthoMCL analysis, we identified a family of GH3-like proteins that is expanded in *C. arabica *(Family 9014; Table 4). GH3 enzymes conjugate amino acids to the auxin indole-3-acetic (IAA), decreasing the concentration of free auxin [89]. This mechanism is important in the regulation of IAA availability in plants. We also detected a family of Aux/IAA proteins that is prominent in *C. arabica *(Family 770; Table 4). Aux/IAA proteins have been shown to function as negative regulators of gene expression mediated by auxin response factor (ARF). A gene similar to auxin receptor TIR1 that promotes ubiquitin (Ub)-mediated degradation of Aux/IAA repressors was identified in *C. arabica *(CaContig 593). In addition, we also detected another putative auxin receptor in *C. arabica*, ABP1 (CaContig16576), a cupin-like protein that is implicated in early auxin responses [90].

Together with small lipophilic "classical phytohormones," small peptides have been described as factors involved in plant growth regulation [91]. Rapid alkalinization factor (RALF) is a small peptide initially isolated in tobacco that induces a rapid alkalinization in cell suspension and inhibits root growth in tomato and Arabidopsis seedlings [92]. Based on BLAST searching, we found a family of RALF peptides in *C. arabica *(two members) and *C. canephora *(five members). However, the evaluation of OrthoMCL families revealed that coffee has a particular family of small peptides slightly similar to RALFs (Family 8498; Table 4). These proteins contain the four cysteines in their C-termini required for RALF activity but are richest in Trp. Further, some members do not contain the conserved dibasic site (Additional File 10), which is essential for processing tomato and Arabidopsis RALFs [92-94]. The isolation and functional analysis of these coffee proteins/peptides constitute an important approach in order to verify whether they exert the same growth retarding effect as RALFs.

### Glycine-Rich Proteins

The glycine-rich protein (GRP) superfamily is a large complex of plant proteins that share the presence of glycine-rich domains arranged in (Gly)n-X repeats [95]. Generally considered as involved in protein-protein interactions, GRPs have diverse functions and structural domains [96]. Evaluating hierarchical clusterization data, we found that several *GRPs *are preferentially expressed in suspension cells treated with BTH, brassinosteroids and NaCl, as well as in embryogenic calli (Additional File 8). Those genes encode GRPs from Class I, which may contain a signal peptide for secretion followed by a glycine-rich region with GGGX repeats [95]. Other *GRPs *(CaContigs 1089, 3317, 10126) were found to be differentially expressed in plantlets and leaves treated with arachidonic acid (Additional File 8). These genes encode proteins containing signal peptides and are similar to class II GRPs, which contain a peptide motif rich in cysteine and tyrosine residues located in their C-termini [95]. However, a deeper annotation revealed that these coffee GRPs contain 12 cysteines instead of the six cysteines of the aforementioned class II GRPs (Additional File 11). These cysteine-rich domain proteins, such as class II AtGRP-3 and NtTLRP, were shown to interact with receptor protein kinase WAK1 [97] and to mediate the cross-linking of proteins to the cell wall [98]. We also detected the presence of some "specific" GRP OrthoMCL families in coffee (Table 4). Family 1231 is composed of class I GRPs, while family 4011 has GRPs from class II that contain six to 10 cysteines (Additional File 11). The diversification of GRPs in coffee is quite remarkable, especially in Class II and is probably important to coffee cell wall dynamics and signal transduction.

### Proteinase Inhibitors (PIs)

The phytocystatins (PhyCys) are 12- to 16-kDa plant proteinaceous inhibitors of Cys-proteases of the papain C1A family [99,100]. All cystatins contain three motifs involved in the interaction with their target enzymes: the reactive site QxVxG, one or two glycine residues in the N-terminal part of the protein, and an A/PW located downstream of the reactive site. In addition, PhyCys contain a consensus sequence ([LVI]-[AGT]-[RKE]-[FY]-[AS]-[VI]-x-[EDQV]-[HYFQ]-N) that conforms to a predicted secondary-helix structure [99]. Family 544 of hypothetical PhyCys was prevalent in coffee plants, containing 21 members in *C. arabica *and six members in *C. canephora *(Table 4). Proteins from family 544 are ≅ 10 kDa, contain a variation of the LARFAV-like domain and do not contain the canonical reactive site QxVxG but have a GG-X-YY motif (Additional File 12). Other OrthoMCL families (2703 and 942) were annotated as containing putative cystatins prevalent in coffee (Table 4; Additional File 12). All members of those three families have low but significant identities (30-40%) with hypothetical cystatins from Arabidopsis (At5g47550), grape (XP_002274494.1) and *Brassica oleracea *(ABD64972). Two *C. canephora *members from those families (CcContigs 7844 and 3825) were highly expressed in leaves from water deficit stressed plants (SH3; Additional File 8; Figure 6A). The majority of these new coffee cystatins do not have signal peptides (Additional File 12), likely being responsible for the regulation of endogenous protein turnover as hypothesized for alfalfa and barley cystatins [101,102]. In a recent phylogenomic analysis, it was proposed that cystatins had undergone a complex and dynamic evolution through gene losses and duplications [103]. This assignment may explain the expansion of cystatins in coffee and may indicate functional diversification of these proteins.

Members of the Potato type II (PotII) inhibitors (Pin2) family are PIs restricted to plants that belong to the MEROPS inhibitor family I20, clan IA [104]. Several Pin2 proteins have a multi-domain structure. However, sequences from coffee-prevalent proteins of OrthoMCL families 7241 and 10273 appear to be uni-domain Pin2 proteins (Additional File 13). Although we did not find any of the coffee *Pin2 *genes preferentially expressed in EST libraries of stressed plants, predicted coffee Pin2 proteins contain signal peptides and, additionally, have 30-40% identity with a Pin2 protein of tobacco that confers tolerance to NaCl and resistance against herbivorous insects in transgenic plants [105]. In addition to the fact that PI expansions may be related to biotic stress regulation, PIs may also have an important role in proteolysis during coffee fruit development because the peptides and amino acids are precursors of coffee flavor and aroma (see below).

### Coffee Cup Quality Related Genes

Coffee cup quality is a complex trait that is being unraveled. The components of coffee endosperm are the source of the precursors of aroma and flavor after roasting. The degradation of sucrose and cell wall polysaccharides generate reducing sugars, which react with amino acids during roasting through Maillard glycation reactions. This reaction gives rise to aromatic products, such as pyrazines, furans and aliphatic acids, which are associated with pleasant flavor and aroma [106]. Conversely, the bitterness of coffee is related to caffeine and chlorogenic acid content in coffee beans [107]. During our annotation, we give a panorama of genes related to coffee cup quality that were, by some means, emphasized in at least one of our bioinformatics analyses.

#### Genes Related to Carbohydrate Metabolism

Due to the importance of the amount and composition of carbohydrates to the final quality of the coffee beverage, the study of coffee bean carbohydrate synthesis and degradation is intense [5-7,108-112]. Coffee bean cell walls are mainly made of galactomannans, arabinogalactans and cellulose [108]. One interesting finding in our analysis was the prevalence of cellulose synthase superfamily proteins (pfam 03552; CesA) in *C. arabica *in relation to *C. canephora *(Figure 5 Additional File 5). CesA proteins interact in a cellulose synthase complex, and it is believed that each cell type contains three types of CesA subunits in a single complex [113]. Therefore, the broader origin of *C. arabica *ESTs may be the reason for the prevalence of *C. arabica *CesAs in comparison to *C. canephora*. The CesA family includes the "true" cellulose synthase genes and eight other families named 'cellulose synthase-like' genes *CslA*-*CslH *[114]. It was verified that some CslA proteins act in the synthesis of mannans and xyloglucans [112,115,116]. The orthologs of these *Csl *genes were found in our *C. arabica *EST data (CaContigs 3405 and 11680).

It is considered that the role of carbohydrates in the differences in cup quality between *C. arabica *and *C. canephora *is related to low molecular weight carbohydrate content, especially sucrose [117]. Arabica grains have a higher amount of sucrose (7.3-11.4%) than *C. canephora *grains (4-5%). Though sucrose is almost completely degraded during coffee bean roasting (0.4-2.8% dry weight), sucrose remains are thought to improve coffee sweetness and cup quality [118]. Privat et al. [6] found that the synthesis of sucrose phosphate synthase (SPS) was higher in late stages of *C. arabica *grains than in *C. canephora*, and invertase activity was lower in Arabica, likely due to the higher expression of invertase inhibitors in this species, justifying the higher sucrose content in *C. arabica *beans. Based on BLAST and OrthoMCL analysis, we found that Invertase Inhibitor 3 (InvI3) is part of a *Coffea *spp.-specific protein family (Family 14814; Table 4). These proteins have 20-30% identity to *Zea mays *invertase inhibitors from the pectin-methylesterase family [6,119,120]. We did not detect *C. arabica *ESTs encoding InvI3, likely due to the low coverage of fruit/seed libraries of this species. The presence of such a particular InvI in coffee may indicate new molecular mechanisms of invertase regulation.

The raffinose family oligosaccharides (RFOs) are soluble galactosyl-sucrose carbohydrates such as raffinose, stachyose and verbascose. Their participation in coffee seed development was assessed by Joet et al. [7], who indicated that RFOs were transiently present during the storage phase and remobilized during mid-stages of development to supply the extensive demand for galactose in galactomannan synthesis. Raffinose synthases (RS; EC 2.4.1.82) catalyze the synthesis of raffinose from sucrose and galactinol [121]. Our CDD-PFAM analysis indicated that *C. arabica *has a larger amount of RS than *C. canephora *(Figure 5). Such data seem to corroborate biochemical analyses that showed that grains from *C. canephora *contain reduced raffinose levels in comparison to Arabica [122,123]. A more careful inspection of RS *C. arabica *clusters revealed that these sequences were derived from diverse tissue libraries. The presence of more EST libraries from stressed plants in *C. arabica *may be the cause of such bias, because RFO accumulation has been associated with responses to abiotic stresses, protecting cellular metabolism from oxidative damage and drought [124,125]. Indeed, a recent analysis indicated that three *C. arabica *RFO synthase transcripts are induced by drought and saline stress (T.B. Santos, I. G. Budzinski, C.J. Marur, C.L. Petkowicz, L.F. Pereira, L.G. Vieira; unpublished results). Therefore, raffinose may exert dual functions in coffee: galactose reservoirs in coffee grains and protective roles in vegetative development.

It is assumed that the RFOs decrease in late stages of coffee bean development are caused by α-D-Galactosidase (α-Gal; EC 3.2.1.22) activity. We identified three α-Gal-encoding genes as more expressed in the late stages of *C. canephora *seed development (CcContigs 2650, 3171, 7083; Additional File 8; Figure 6A), data that agree with previous findings verifying increased α-Gal activity during *in vitro *germination of coffee beans [126]. Together with α-Gal, β-mannosidases (EC 3.2.1.25) and Endo β-mannanase (EC 3.2.1.78) are enzymes involved in the degradation of galactomannans during germination of seeds. Despite the fine analysis of *C. arabica *β-mannanases and α-Gal [109,126], there is no biochemical analysis of β-mannosidases activity in coffee of which we are aware. We found that β-mannosidases are preferentially expressed in germinating seeds of *C. arabica and C. canephora *(CaContig 3009, CcContig6678; Figure 6; Additional File 8), a similar pattern in comparison to α-Gal from *C. canephora *(CcContig 6678; Additional File 8).

#### Amino Acid Content: Storage Protein Synthesis and Protease Expression

As cited above, proteins and amino acids are also fundamental for the generation of flavor and aroma-related Maillard-end products. In effect, the level of protein synthesis during early fruit stages, the amount of seed storage proteins (SSPs) in the endosperm and the relationship between proteinases and their inhibitors during seed development are all factors that determine the amino acid content in mature beans. Examining the expression profile of the SE2 library, we found a series of ribosomal proteins expressed in this stage of seed maturation (Figure 6A; Additional File 8), indicating an intense cellular effort in translation. Many SSPs are enriched in cysteines, which confer high stability to these proteins, an important factor for storage proteins. These cysteines are also a source of sulfur used in seed germination. Two genes involved in cysteine metabolism, protein folding and sulfur metabolism were preferentially expressed in the early stage of *C. canephora *seed maturation (SE2 library; Figure 6A). CcContigs 7827 and 99 encode a cysteine synthase (O-acetylserine (thiol) lyase) (EC 4.2.99.8), an enzyme that synthesizes cysteine [127], and a protein disulfide isomerase (PDI), an enzyme that catalyzes the formation and breakage of disulfide bonds between cysteine residues within proteins as they fold [128], respectively.

In coffee, the Cupin family protein 11 S globulin represents 45% of the total protein in the endosperm (corresponding to 5-7% of coffee bean dry weight) [129] and is probably one of the main sources of nitrogen during coffee bean roasting. Our expression hierarchical clustering analysis indicated that two 11 S globulin genes were preferentially expressed in *C. arabica *fruit libraries (CaContigs 12252 and13966; Additional File 8), and one was more highly expressed in the late stages of *C. canephora *seed development (i.e., 42 weeks after pollination) (CcContig 4069; Additional File 8). This contig was the second most abundant in the *C. canephora *database (Additional File 7) after a 2 S albumin (CcContig1385; Additional File 7). We also identified a cysteine and an aspartic protease preferentially expressed in the last phase of Arabica seed maturation (CaContigs 7768 and 8165; Additional File 8). The coincidence of expression profiles of important storage proteins such as 11 S globulin and 2 S albumin together with proteinases is an indication that the release of free amino acids or small peptides that contribute to coffee cup quality can occur in the final stage of coffee maturation.

#### Secondary Metabolism: Caffeine, Trigoneline and Chlorogenic Acid

Other precursors of flavor and aroma in coffee are secondary metabolites, such as alkaloids (caffeine and trigoneline) and phenylpropanoid chlorogenic acid (CGA). These three components, together with sucrose, seem to be the main factors influencing coffee quality, because sucrose and trigoneline enhance coffee quality, while CGA and caffeine confer bitter taste [7,107,130-133]. The comparison between the two coffee species showed that *C. arabica *has more trigoneline and sucrose, and *C. canephora *contains more CGA and caffeine [131]. Despite intense annotation, our data did not reveal any outstanding results concerning the differential expression of the genes in the metabolic pathways of these compounds during fruit development or any interesting difference between *C. arabica *and *C. canephora *plants.

## Conclusion

We assembled ESTs from *C. arabica *and *C. canephora *and applied a diverse array of bioinformatics tools to extract information about gene content features, transcriptome changes and novel genes and gene families. The results concerning the prevalence of proteins related to sugar metabolism in *C. arabica *and signal transduction in *C. canephora *can be correlated with agronomical characteristics of each species due to the better cup quality of *C. arabica *and the high tolerance to specific stresses in *C. canephora *plants. Despite knowing that comparisons between these *Coffea *species data should be carefully inspected, our initiative established possible transcriptomic elements that could guide the coffee scientific community in unraveling the molecular mechanisms that distinguish these two extremely important *Coffea *species. In addition, the annotation of coffee-specific/prominent genes adds new elements to genomic initiatives that are searching for traits that could differentiate coffee from other Asteridae species. In a recent report, Vidal et al. [30] showed that *C. arabica *displays differential expression of homeologous genes and suggested that *C. arabica *ancestral subgenomes encode proteins involved in different physiological mechanisms, adding a new element of investigation concerning gene expression regulation in coffee plants.

All data presented here are available at http://www.lge.ibi.unicamp.br/coffea. We believe that such data are a valuable aid to the interpretation of coffee development, providing insights that could help coffee breeding programs and indicating potential targets for functional analysis and biotechnology products of such socially and economically important species.

## Methods

### EST assembly and trimming

ESTs from *C. arabica *(187,142) and *C. canephora *(78,470) were derived from 43 libraries collected by the BCGP and from 8 libraries of *C. canephora *EST sequencing initiative of the Nestlé Research Center (8). The Brazilian project sources were mainly two *C. arabica *genotypes (Catuai and Mundo Novo, with the exception of germinating seeds from cv. Rubi) and one *C. canephora *genotype (Conillon). The Cornell-Nestlé project EST sources were five different varieties of *C. canephora *[22]. Sequences were trimmed using BDTrimmer to remove ribosomal sequences, polyA/T tails, low quality sequences, vector sequences (UniVec database) and *E. coli *contaminants [134]. EST assembling was executed using the CAP3 program, with a minimum similarity threshold of 90% and a minimum overlap of 40 bases. ESTs from each species were assembled separately, and the genotypes were assembled together into the same species. After the assembly, nucleic acid contamination from bacterial organisms that were not removed during trimming analysis (putative endophytes of coffee) was detected using BLASTN against a version of the NT database containing only bacteria (NT-bac) and BLASTX against the NR database. The results against NT-bac with E-values > 1e^-40 ^and the percent of identical nucleotides > 80% were considered bacterial contamination. In addition, hits against NR with a percent of identity > 30% and all of the hits against bacteria were considered bacterial contamination. All of the BCGP ESTs were submitted to GenBank with accession numbers GT640310-GT640366, GT669291-GT734396, GW427076 - GW492625 (*C. arabica*) and GT645618-GT658452 (*C. canephora*).

### Single Nucleotide Polymorphism (SNP) analyses and GC content

QualitySNP [24] was used to analyze polymorphisms present in *C. arabica *and *C. canephora*. QualitySNP uses three quality filters for the identification of reliable SNPs. The first filter screens for all potential SNPs. False SNPs caused by sequencing errors are identified by the chromatogram quality given by Phred. The second filter is the core filter, which uses a haplotype-based strategy to detect reliable SNPs. The clusters with potential paralogs are identified using the differences in SNP number between potential haplotypes of the same contig. All potential haplotypes consisting of only one sequence are removed, and singleton SNPs that are not linked to other polymorphisms are not considered. This may lead to an underestimation of nucleotide diversity but assures that false positives will be discarded. The last filter screens SNPs by calculating a confidence score based on sequence redundancy and base quality. To label each polymorphism as synonymous or non-synonymous, the correct open reading frame (ORF) of each sequence was identified by looking for similarity calculated with the FASTA algorithm against the Uniprot databank http://www.uniprot.org using an E-value threshold of -05. The alignments were analyzed with QualitySNP script GetnonsySNPfasty, which corrects frame shifts and attempts to expand the 3' end until the next stop codon and the 5' end until the next ATG codon. This script identifies if the polymorphism changes the amino acid, labeling each polymorphism as non-synonymous (KA) or synonymous (KS). This information was used to calculate KA/KS ratios for positive selection using kaks calculator software [135]. All of the ORFs predicted in QualitySNP were used to calculate the GC content of *C. arabica *and *C. canephora*. A total of 1,380 full length sequences > 200 bp of *Arabidopsis thaliana *were extracted from Genbank. Sequences of *Solanum lycopersicum *were also randomly retrieved from the Kazusa http://www.kazusa.or.jp/jsol/microtom/indexj.html and SGN databanks [23]. Total GC and GC3 were calculated for each sequence and plotted in a histogram graph with 100 classes, which were smoothed by using the average of each three sets of classes.

### Automatic Functional Annotation, Metabolic Pathways and Evaluation of Protein Domains

The complete set of ESTs from *C. arabica *and *C. canephora *were automatically annotated using the AutoFACT program [31]. AutoFACT summarizes results of BLAST similarity searches against nucleotide, protein and domain databases in functional annotation. The databases used were Uniref100, Uniref90, NCBI-nr, KEGG and CDD (E-value ≤ 1E-5). The annotation was submitted to the Pathologic module of the Pathway Tools program (version 13.0) in order to generate metabolic maps. Pathologic module looks at the product name and E.C. number of annotations and imports the pathways likely to be present from the reference database (MetaCyc). The *C. arabica *and *C. canephora *metabolic maps were compared with PlantCyc, which contains curated information about pathways present in > 250 plant species. The divergence among the maps was manually annotated to eliminate false positives. To evaluate protein domains, ESTs were submitted to similarity searches against the CDD-PFAM database using RPS-BLAST (E-value ≤ 1e^-10^). Data were normalized by dividing the number of clusters from each CDD-PFAM by the total number of hits from each species against CDD-PFAM.

### Gene Ontology Analyses

Coffee datasets were annotated and mapped for the gene ontologies "Biological Process" and "Molecular Function" (only level 3) by Blast2go [43]. Blast2go lists all gene ontology terms found in biological processes and molecular functions found in each dataset and associates the amount of sequences with each term. These data were normalized to the total number of sequences that were labeled with a gene ontology term. Hypergeometric distribution statistical analysis [44] was applied in the datasets from fruit and leaf to find the sub- and over-estimated GO terms in each species.

### Orthologous Clustering (Ortho-MCL)

The Ortho-MCL algorithm [47] was applied to generate orthologous groupings. Two different datasets were used: i) the annotated proteins from the available complete genomes of *A. thaliana *(27,379 proteins), *O. sativa *(56,797 proteins), *Ricinus communis *(31,221 proteins) and *Glycine max *(66,210 proteins) and ii) the proteins predicted by FrameDP software [48] from the available EST assemblies for *C. arabica *(28,585 predicted proteins), *C. canephora *(16,477 predicted proteins) and *S. lycopersicum *(52,437 predicted proteins). All proteins were compared (all against all) using BLASTP, and a score for each pair of proteins (u, v) with significant BLAST hits was assigned (E-value 1e^-5^; with at least 50% of similarity). Based on these scores, the MCL algorithm was applied to find clusters in this graph. The protocol used is described at http://lge.ibi.unicamp.br/Ortho_MCL_UserGuide.txt.

### Gene Expression Hierarchical Clustering Analysis

For *in silico *expression analysis, contig and singlet frequencies across the libraries were obtained from the dataset derived from the CAP3 assembly. The frequency of a contig over a library represents its transcript abundance. Only contigs containing more than two ESTs were used for transcript profiling. Differentially expressed contigs were identified using two statistical tests, R [55] and AC [56], with the webtool IDEG6 [57]. In R statistics, a threshold p-value of 0.05 (95% confidence) was used with Bonferroni correction. AC statistics were calculated for pairwise combinations of all libraries. Under this criterion, a contig was considered of significant interest if the AC statistics of at least one library against all of the other libraries were lower than the threshold 0.05. The resulting differentially expressed contigs were obtained with the union of the two sets above. Each library frequency was then normalized by the frequency of the contig.

In an attempt to cluster elements that are similar (in some sense), hierarchical clustering [136] of the differentially expressed contigs was performed using MatlabR2009a (The Mathworks). Hierarchical algorithms attempt to group the differentially expressed contigs based on the expression profile of these contigs in the libraries. The clustering of the rows (contigs) was performed, generating a heat map and a dendrogram. The libraries were manually sorted according to tissue sources and stress conditions, visually creating two libraries groups: "development" libraries and "stress" libraries.

## Authors' contributions

JMCM: ESTs and cluster re-annotations, conception of bioinformatics analyses, evaluation and interpretation of GC content, Ortho-MCL, CDD-PFAM, Gene Ontology and Hierarchical clustering data, conception and writing of the manuscript; ROV: EST assembly, conception of bioinformatics analyses, GC content and SNP analyses and evaluation, Gene Ontology and Ortho-MCL analysis; MFC: EST assembly, conception of bioinformatics analyses and CDD-PFAM analysis; EKT: Hierarchical clustering analysis; LPP: AutoFACT and Metabolic Pathways analysis; GGLC: EST assembly and Ortho-MCL analysis; LFPP: SNP evaluation and revision of the manuscript; ACA: Coordination of EMBRAPA EST libraries and revision of the manuscript; CAC: Coordination of AEG/FAPESP EST libraries and revision of the manuscript; LGEV: Coordination of the EST consortium and revision of the manuscript; GAGP: coordination of the bioinformatics group and elaboration of the final manuscript. All authors read and approved the final manuscript.

## Supplementary Material

Additional file 1 **Description of Brazilian Coffee Genome Project ESTs and cDNA libraries**. Word file describing the methods used for the production of Brazilian coffee ESTs libraries, the tissues and experimental conditions used. Click here for file

Additional file 2 **Number of contigs composed from sequence originated from one or more libraries**. TIF file containing a figure depicting the correlation between number of contigs vs number of libraries. Click here for file

Additional file 3 **Global data of SNP detection in *C. arabica *and *C. canephora *EST datasets**. Word file containing an overall analysis of SNPs from *C. arabica *and *C. canephora *contigs. Click here for file

Additional file 4 **Annotation of KA/KS ratio in *Coffea *spp. contigs**. Word file containing the annotation of Top 20 *C. arabica *contigs with highest and lowest KA/KS ratio (A); Annotation of Top 20 *C. canephora *contigs with highest and lowest KA/KS ratio (B). ID: Contig number; KS: rate of synonymous substitutions, KA: rate of non-synonymous substitutions; KA/KS: KA to KS ratio; First Hit (BLASTX-NR): Most similar sequence in GenBank; E-value: E-value of most similar sequence; Annotation: automatic annotation based in AutoFACT results. Click here for file

Additional file 5 **Top 20 *Coffea *spp. PFAM families**. Word file containing the ranking of PFAM families in *C. arabica *and *C. canephora*. Click here for file

Additional file 6 **Annotation of Top 20 genes with the widest distribution among *Coffea *spp. cDNA libraries**. Word file containing the ranking of genes distributed throughout coffee EST libraries. ID: Contig number; #: number of libraries represented in each contig; #ESTs: number of ESTs that compose each contig; First Hit (BLASTX-NR): Most similar sequence in GenBank; E-value: E-value of most similar sequence; Annotation: automatic annotation based in AutoFACT results. Click here for file

Additional file 7 **Annotation of 20 genes with the highest expression among *Coffea *spp. cDNA libraries**. Word file containing the ranking of genes more expressed in coffee EST libraries. ID: Contig number; #: number of libraries represente in each contig; #ESTs: number of ESTs that compose each contig; First Hit (BLASTX-NR): Most similar sequence in GenBank; E-value: E-value of most similar sequence; Annotation: automatic annotation based in AutoFACT results. Click here for file

Additional file 8 **Annotation of selected differentially expressed genes in coffee EST libraries according to hierarchical clustering analysis**. Excel file containing the annotation of genes with differential expression profile in coffee EST libraries according to hierarchical clustering analysis. Worksheet CA: *C. arabica *contigs; Worksheet CC: *C. canephora *contigs. Libraries: Tissues and organs used in the libraries construction; Nomenclature: code of the library; Contig ID: Contig number; Annotation: automatic annotation based in AutoFACT results. Click here for file

Additional file 9 **Results concerning some genes related with drought stress (Dehydrins, LEAs and Metallothioneins)**. Word file describing results and a brief discussion about dehydrins, LEAs and Metallothioneins expressed in coffee EST libraries. Click here for file

Additional file 10 **RALF and RALF-like peptides**. Word file containing the sequences of RALF and RALF-like peptides expressed in coffee. In magenta: dibasic sites; in yellow: cysteine residues. Click here for file

Additional file 11 **OrthoMCL families of Glycine Rich Proteins (GRP)**. Word file containing the sequences of Glycine Rich Proteins expressed in coffee. In yellow: cysteine residues; Underlined: signal peptide for secretion. Click here for file

Additional file 12 **OrthoMCL families of Cystatins**. Word file containing the sequences of Cystatins expressed in coffee. In green: variation of LARFAV motif; in yellow: new motif GG-X-YY; in blue: QVVAG motif. Click here for file

Additional file 13 **OrthoMCL families of PinII Serine Proteinase Inhibitors**. Word file containing the sequences of PinII Serine Proteinase Inhibitors in coffee. Click here for file
