# Supplementary material for: An EST-based analysis identifies new genes and reveals distinctive gene expression features of Coffea arabica and Coffea canephora
Source: BMC Plant Biol. 2011 Feb 8;11:30. doi: 10.1186/1471-2229-11-30 (PMC3045888; doi:10.1186/1471-2229-11-30)
Supplement: Additional file 1 — Description of Brazilian Coffee Genome Project ESTs and cDNA libraries. Word file describing the methods used for the production of Brazilian coffee ESTs libraries, the tissues and experimental conditions used. [file 1471-2229-11-30-S1.PDF]

Additional File I: Description of Brazilian coffee ESTs and description of libraries

A) Confection of Brazilian initiative cDNA libraries

RNA from coffee tissues was extracted from different developmental stages and from plant suffering different stress conditions. Poly(A)+ RNA was purified from total RNA using the Oligotex Kit (Qiagen, USA). cDNA libraries were constructed using the SuperScript Plasmid System and Plasmid Cloning Kit (Invitrogen, USA) with about 1-2 µg poly(A)+ RNA. The efficiency of cDNA synthesis was monitored with radioactive nucleotides. cDNA were size fractionated on a Sepharose CL-2B column. Aliquots of each fraction were eletrophoresed in agarose gel to determine the size range of cDNAs. Fractions containing cDNA larger than 500 bp were ligated into pSPORT1 and pSPORT6 vectors (Invitrogen) at the SalI-NotI site. The resulting plasmids were transformed in *E. coli* DH10B or DH5α cells (Invitrogen) by electroporation. Plasmid DNA was purified using a modified alkaline lysis method (Sambrook et *al.,* 1989). Sequencing reactions were conducted using the ABI BigDye Terminator Sequencing kit (Applied Biosystems). cDNA inserts were sequenced from the 5’ end with T7 promoter primer (5’-TAATACGACTCACTATAGGG-3’) or M13 Rev in the pSPORT1 vector with SP6 primer (5’-ATTTAGGTGACACTATAG-3’) in the pSPORT6. Sequencing reaction products were analyzed on ABI 3700 sequencers (Applied Biosystems).

B) Description of the coffee ESTs libraries

Coffea arabica	**Library**	**Description**	**Cultivar**	**Source**
	AR1	Leaves treated with araquidonic acid	Mundo novo	Brazil
	LP1	Plantlets treated with araquidonic acid	Mundo novo + Catuai	Brazil
	CB1	Suspension cells treated with benzothiadiazole and brassinoesteroids	Catuai	Brazil
	CL2	Hypocotyls treated with benzothiadiazole	Mundo novo + Catuai	Brazil
	EA1, IA1, IA2	Embryogenic calli	Catuai	Brazil
	EB1	Zygotic embryo	Mundo novo + Catuai	Brazil
	EM1, SI3	Germinating seeds (whole seeds and zygotic embryos)	Catuai	Brazil
	FB1, FB2, FB4	Flower buds in different developmental stages	Mundo novo	Brazil
	FR1, FR2	Flower buds + pinhead fruits + fruits at different stages	Mundo novo	Brazil
	CA1	Non embryogenic calli	Mundo novo + Catuai	Brazil
	IC1	Non embryogenic calli	Catuai	Brazil
	PC1	Non embryogenic calli + 2,4-D	Mundo novo + Catuai	Brazil
	LV4, LV5	Young leaves from orthotropic branch	Mundo novo	Brazil
	LV8, LV9	Mature leaves from plagiotropic branches	Mundo novo	Brazil
	NS1	Roots infected with nematodes	Mundo novo + Catuai	Brazil
	PA1	Primary embryogenic calli	Mundo novo + Catuai	Brazil
	RM1	Leaves infected with leaf miner and coffee leaf rust	Mundo novo	Brazil
	RT3	Roots	Mundo novo	Brazil
	RT5	Roots with benzothiadiazole	Mundo novo	Brazil
	RT8	Suspension cells with stressed with aluminum	Catuai	Brazil
	RX1	Stems infected with *Xylella* spp	Catuai	Brazil
	SH2	Water deficit stresses field plants (pool of tissues)	Catuai	Brazil
	SS1	Well-watered field plants (pool of tissues)	Catuai	Brazil
	CS1	Suspension cells with mannose Nacl and KCL	Catuai	Brazil
	BP1	Suspension cells treated with acibenzolar-S-methyl	Catuai	Brazil
	PL1	?	?	Brazil
	SI1	Germinating seeds	Rubi	Brazil
	SI2	Germinating seeds	Rubi	Brazil
	CD1	Suspension cells	Catuai	Brazil
	CL1	Suspension cells	Catuai	Brazil
	CM1	?	Mundo novo + Catuai	Brazil
	LM3	?	Mundo novo + Catuai	Brazil
	RT7	Root	Mundo novo + Catuai	Brazil
	FB3	Flower buds	Mundo novo	Brazil
	FP2	?	Mundo novo + Catuai	Brazil
Coffea canephora	**Library**	**Description**	**Cultivar/ Varieties**	
	LF1	Young leaves,	BP409	Nestlé
	PP1	Pericarp, all developmental stages	BP358,BP409,BP42, BP961,Q121	Nestlé
	SE1	Whole cherries,18 and 22 week after pollination	BP358,BP409, BP42,Q121	Nestlé

	SE2	Whole cherries,18 and 22 week after pollination	BP358,BP409, BP42,Q121	Nestlé
	SE3	Endosperm and perisperm, 30 week after pollination	BP409,BP961,Q121	Nestlé
	SE4	Endosperm and perisperm, 42 and 46 weeks after pollination	BP358,BP409,BP42, BP961,Q121	Nestlé
	EC1	Embriogenic calli	Conilon	Brazil
	SH1	Leaves from water deficit stressed plants	Conilon	Brazil
	SH3	Leaves from water deficit stressed plants (drought resistant clone)	Conilon	Brazil
