# Supplementary figures and images for: An EST-based analysis identifies new genes and reveals distinctive gene expression features of Coffea arabica and Coffea canephora
Source: BMC Plant Biol. 2011 Feb 8;11:30. doi: 10.1186/1471-2229-11-30 (PMC3045888; doi:10.1186/1471-2229-11-30)

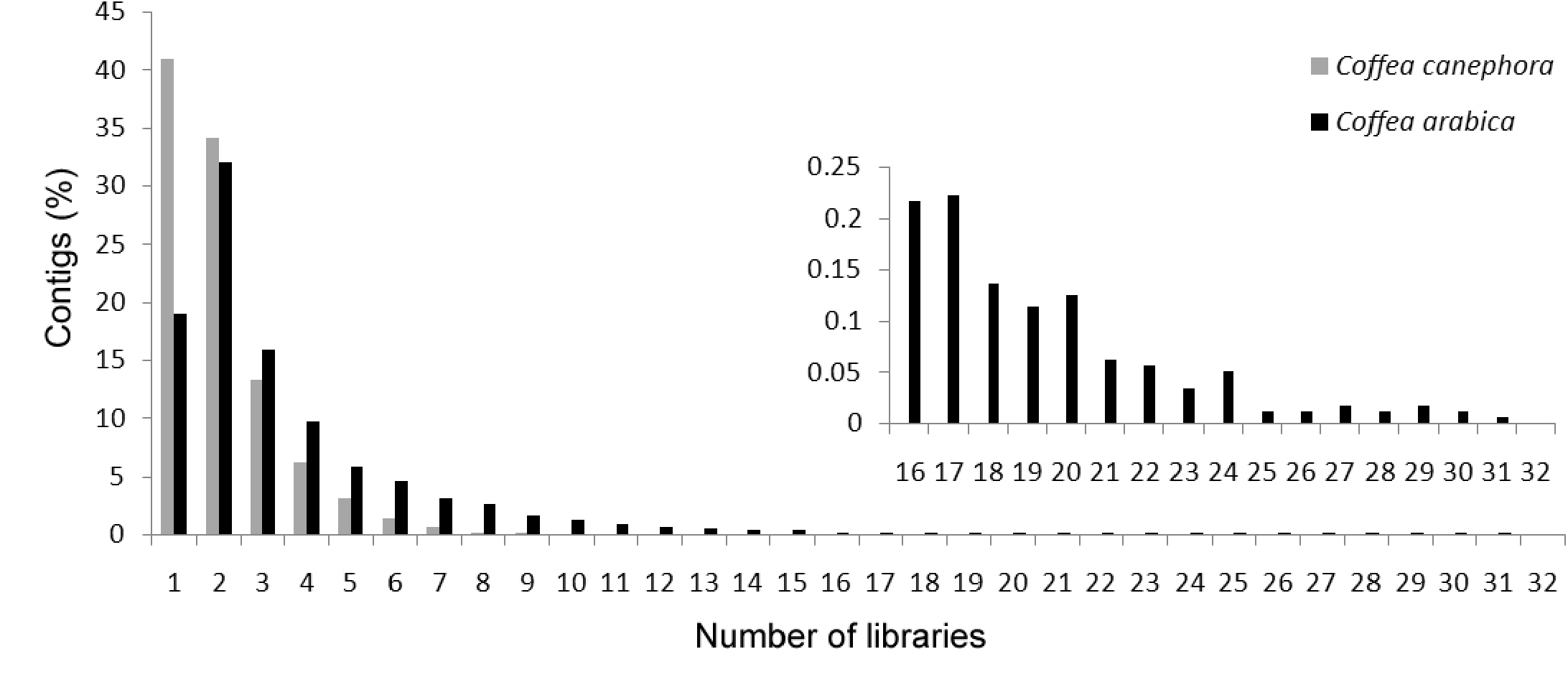

Supplement: Additional file 2 — Number of contigs composed from sequence originated from one or more libraries. TIF file containing a figure depicting the correlation between number of contigs vs number of libraries. [file 1471-2229-11-30-S2.TIFF]
