# Supplementary material for: An EST-based analysis identifies new genes and reveals distinctive gene expression features of Coffea arabica and Coffea canephora
Source: BMC Plant Biol. 2011 Feb 8;11:30. doi: 10.1186/1471-2229-11-30 (PMC3045888; doi:10.1186/1471-2229-11-30)
Supplement: Additional file 3 — Global data of SNP detection in C. arabica and C. canephora EST datasets. Word file containing an overall analysis of SNPs from C. arabica and C. canephora contigs. [file 1471-2229-11-30-S3.PDF]

Additional File 3: Global data of SNP detection in *C. arabica* and *C. canephora* EST data.

Coffea arabica		**CT***	**AG**	**AT**	**AC**	**TG**	**CG**
Total Contigs with SNPs	4,535						
True SNPs	18,390						
Transitions	10,142	5,234	4,908	-	-	-	-
Transversions	6,078	-	-	1,573	1,325	1,493	1,687
Indels	2,126						
Frequency Transitions	55.28%	51.61%	48.39%				
FrequencyTransversions	33.13%			25.88%	21.80%	24.56%	27.76%
Frequency Indels	11.59%						
Tri-allelic Pol	44						
Tetra-allellic Pol	0						
Total length	5,121,760						
Frequency of SNPs+Indels	0.35906						
Frequency SNPs	0.31669						
Frequency INDELs	0.04151						
SNPs+Indels per Contig	4.05513						
SNPs per Contig	3.57663						
INDELS per Contig	0.46880						
# Haplotypes	11,288						
Haplotypes/Contig	2.489085						
Coffea canephora		**CT**	**AG**	**AT**	**AC**	**TG**	**CG**
Total Contigs with SNPs	2,000						
True SNPs	4,724						
# Transitions	2,384	1,211	1,173	-	-	-	-
# Transversions	1,588	-	-	358	356	468	406
# Indels	727						
% Transitions	50.73%	50.80%	49.20%				
% Transversions	33.79%			22.54%	22.42%	29.47%	25.57%
% Indels	15.47%						
Tri-allelic Pol	25						
Tetra-allellic Pol	0						
Total length	2,077,254						
Frequency SNPs+Indels	0.22742						
Frequency SNPs	0.19121						
Frequency INDELs	0.03500						
SNPs+Indels per Contig	2.36200						
SNPs per Contig	1.98600						
INDELS per Contig	0.36350						
# Haplotipes	5,360						
Haplotipes/Contig	2.68						

*The dinucleotides described indicate the direction of putative mutations (i.e., CT = cytosine to thymine).
