# Supplementary material for: An EST-based analysis identifies new genes and reveals distinctive gene expression features of Coffea arabica and Coffea canephora
Source: BMC Plant Biol. 2011 Feb 8;11:30. doi: 10.1186/1471-2229-11-30 (PMC3045888; doi:10.1186/1471-2229-11-30)
Supplement: Additional file 4 — Annotation of KA/KS ratio in Coffea spp. contigs. Word file containing the annotation of Top 20 C. arabica contigs with highest and lowest KA/KS ratio (A); Annotation of Top 20 C. canephora contigs with highest and lowest KA/KS ratio (B). ID: Contig number; KS: rate of synonymous substitutions, KA: rate of non-synonymous substitutions; KA/KS: KA to KS ratio; First Hit (BLASTX-NR): Most similar sequence in GenBank; E-value: E-value of most similar sequence; Annotation: automatic annotation based in AutoFACT results. [file 1471-2229-11-30-S4.PDF]

Additional File 4: Annotation of KA/KS ratio in *Coffea* spp. contigs.

A): Annotation of Top 20 *C. arabica* contigs with highest and lowest KA/KS ratio

**High KA/KS**						
Sequence	KS	KA	KA/KS	Firts Hit (BlastX-NR)	E-value	Annotation
Contig9578	0.004	0.0094	2.0952	emb|CAO71103.1| unnamed protein product [*Vitis vinifera*]	2.00E-62	Major intrinsic protein (MIP) superfamily
Contig4156	0.003	0.0064	2.0936	ref|NP_568215.2| SNG2 (Sinapylglucose accumulator 2)[*Arabidopsis thaliana*]	1.00E-133	Serine carboxypeptidase-like
Contig1735	0.006	0.0127	2.0609	gb|ABK94488.1| unknown [*Populus trichocarpa*]	5.00E-81	Glutathione peroxidase
Contig12903	0.006	0.0113	1.8721	emb|CAA46808.1| Rieske FeS [*Nicotiana tabacum*]	1.00E-103	Cytochrome b6-f complex iron-sulfur
Contig15568	0.002	0.0037	1.8685	emb|CAO45533.1| unnamed protein product [*Vitis vinifera*]	0.00E+00	Leucine-rich repeat transmembrane protein kinase
Contig6193	0.018	0.0341	1.8471	gb|ABK91930.1| Mal d 1 isoallergen [Malus x domestica]	7.00E-45	Major allergen Mal d/ PR10-like proteins
Contig9214	0.006	0.0102	1.8168	emb|CAO65210.1| unnamed protein product [*Vitis vinifera*]	5.00E-57	Jasmonate ZIM-domain protein 1
Contig5255	0.009	0.0158	1.7112	gb|ABK91930.1| Mal d 1 isoallergen [Malus x domestica]	1.00E-44	Major allergen Mal d/PR10-like proteins
Contig10695	0.022	0.0349	1.5664	gb|AAX49391.1| OLE-3 [*Coffea canephora*]	4.00E-61	Oleosin
Contig17112	0.003	0.0052	1.5054	emb|CAO40012.1| unnamed protein product [*Vitis vinifera*]	1.00E-152	Beta-glucosidase
Contig2205	0.004	0.0064	1.4983	gb|AAP42136.1| erg-1 [*Solanum tuberosum*]	1.00E-121	Phosphate-responsive protein (phi-1)
Contig1918	0.008	0.012	1.4876	emb|CAO45507.1| unnamed protein product [*Vitis vinifera*]	1.00E-69	Protein phosphatase 2C-like protein
Contig82	0.008	0.0116	1.4828	emb|CAO17977.1| unnamed protein product [*Vitis vinifera*]	8.00E-71	Vegetative storage protein
Contig7582	0.013	0.0189	1.4709	emb|CAO40168.1| unnamed protein product [*Vitis vinifera*]	8.00E-52	PII protein
Contig2035	0.004	0.0059	1.4164	gb|AAP40022.1| callus-expressing factor [*Nicotiana tabacum*]	1.00E-97	Ethylene-responsive element binding protein ERF2
Contig2469	0.007	0.0092	1.3556	dbj|BAB09523.1| unnamed protein product [*Arabidopsis thaliana*]	5.00E-58	Cytochrome b5 domain-containing protein
Contig2994	0.008	0.0102	1.3395	gb|ABK92934.1| unknown [*Populus trichocarpa*]	4.00E-82	Coated vesicle membrane protein
Contig2672	0.006	0.0076	1.3247	gb|ABK92454.1| unknown [*Populus trichocarpa*]	3.00E-98	5'-Methylthioadenosine Nucleosidase
Contig8174	0.004	0.0055	1.3046	dbj|BAA03526.1| F1-ATPase gammma subunit [*Ipomoea batatas*]	1.00E-137	F1-ATPase gammma subunit
Contig16950	0.02	0.0263	1.2831	gb|ABG73415.1| chloroplast pigment-binding protein CP29 [*Nicotiana tabacum*]	1.00E-92	Chlorophyll A-B binding protein CP29
**Low KA/KS**						
Sequence	KS	KA	KA/KS	Firts Hit (BlastX-NR)	E-value	Annotation
Contig6524	0.057	0.0017	0.0298	dbj|BAD10939.1| 14-3-3 protein [*Nicotiana tabacum*]	9.00E-133	14-4-3 protein
Contig2240	0.027	0.001	0.0354	gb|EAZ34301.1| hypothetical protein OsJ_017784 [*Oryza sativa* (japonica cultivar-group)]	0.00E+00	Tubulin beta-2 chain
Contig15974	0.028	0.001	0.036	emb|CAO23450.1| unnamed protein product [*Vitis vinifera*]	1.00E-172	Male sterility protein 2/ acyl CoA reductase
Contig5581	0.025	0.0009	0.0363	emb|CAO15686.1| unnamed protein product [*Vitis vinifera*]	0.00E+00	Rubisco activase
Contig15938	0.028	0.0011	0.038	emb|CAA81527.1| S-adenosyl-L-homocysteine hydrolase [*Catharanthus roseus*]	0	S-adenosyl-L-homocysteine hydrolase
Contig4350	0.038	0.0015	0.0406	emb|CAO44494.1| unnamed protein product [*Vitis vinifera*]	1.00E-134	Light-harvesting complex II protein 5
Contig13838	0.021	0.0009	0.0411	emb|CAO66235.1| unnamed protein product [*Vitis vinifera*]	0.00E+00	Catalase
Contig5884	0.047	0.002	0.0415	gb|AAD56018.1|60S ribosomal protein L10 [*Vitis riparia*]	1.00E-123	60S ribosomal protein L10
Contig2627	0.065	0.0027	0.0416	gb|AAS48586.1| eukaryotic initiation factor 5A2 [*Capsicum annuum*]	2.00E-72	Eukaryotic translation initiation factor 5
Contig11187	0.016	0.0006	0.0417	emb|CAA42660.1| luminal binding protein (BiP) [*Nicotiana tabacum*]	0	Luminal-binding protein
Contig3104	0.033	0.0014	0.0433	gb|EAZ04358.1| hypothetical protein OsI_025590 [Oryza sativa (indica cultivar-group)]	1.00E-107	Putative secretory carrier-associated membrane protein 1
Contig6370	0.039	0.0017	0.0441	emb|CAN79984.1| hypothetical protein [*Vitis vinifera*]	1.00E-124	LHCA4 (Photosystem I light harvesting complex gene 4)

Contig12505	0.023	0.001	0.045	gb|ABV80356.1| phosphoenolpyruvate carboxylase [*Gossypium hirsutum*]	0	Phosphoenolpyruvate carboxylase
Contig6753	0.057	0.0025	0.0451	emb|CAO66090.1| unnamed protein product [*Vitis vinifera*]	1.00E-64	Iron-sulfur cluster assembly complex protein
Contig1269	0.018	0.0008	0.0456	gb|ABP98813.1| chloroplast biotin carboxylase [*Gossypium hirsutum*]	0.00E+00	Biotin carboxylase
Contig8981	0.023	0.001	0.0458	emb|CAO22101.1| unnamed protein product [*Vitis vinifera*]	1.00E-173	60S ribosomal protein L4/L1
Contig4659	0.024	0.0011	0.0465	emb|CAN80621.1| hypothetical protein [*Vitis vinifera*]	1.00E-139	Beta-1.3-glucanase
Contig9099	0.031	0.0015	0.0475	ref|NP_177596.1| NRP1 (NAP1-RELATED PROTEIN 1) [*Arabidopsis thaliana*]	1.00E-90	NRP1 (Nap1-related protein 1)
Contig4086	0.014	0.0007	0.0476	emb|CAO61278.1| unnamed protein product [*Vitis vinifera*]	0	Leucine Rich Repeat family protein
Contig3271	0.041	0.002	0.0484	emb|CAO71073.1| unnamed protein product [*Vitis vinifera*]	1.00E-81	Glutamate binding protein

ID: Contig number; KS: rate of synonymous substitutions, KA: rate of non-synonymous substitutions; KA/KS: KA to KS ratio; First Hit (BLASTX-NR): Most similar sequence in GenBank; E-value: E-value of most similar sequence; Annotation: automatic annotation based in AutoFACT results.

B) Annotation of Top 20 *C. canephora* contigs with highest and lowest KA/KS ratio

**High KA/KS**						
Sequence	KS	KA	KA/KS	Firts Hit (BlastX-NR)	E-value	Annotation
Contig2864	0.0069	0.00905	1.30796	gb|AAT40548.1| Putative vicilin, identical [*Solanum demissum*]	1.00E-151	Vicilin/ globulin
Contig386	0.0047	0.0061	1.2914	gb|AAL35365.1| ascorbate peroxidase [*Capsicum annuum*]	1.00E-134	Ascorbate peroxidase
Contig3937	0.0061	0.00749	1.23655	gb|ABK93197.1| unknown [*Populus trichocarpa*]	3.00E-54	Membrane steroid-binding protein
Contig2694	0.0163	0.01959	1.20048	gb|AAF31403.1| putative glycine-rich RNA binding protein 3 [*Catharanthus roseus*]	3.00E-38	Glycine-rich RNA binding protein-like
Contig1112	0.0038	0.00446	1.18711	gb|ABK95575.1| unknown [*Populus trichocarpa*]	1.00E-170	Aminopeptidase N
Contig3653	0.0039	0.00446	1.13298	gb|AAQ94896.1| putative N-methyltransferase [*Coffea canephora*]	0.00E+00	Dimethylxanthine Methyltransferase
Contig6678	0.0032	0.00362	1.13002	gb|AAL37719.1|AF413204_1 beta-mannosidase [*Solanum lycopersicum*]	0	Beta-mannosidase enzyme
Contig175	0.0029	0.00311	1.07339	emb|CAO24398.1| unnamed protein product [*Vitis vinifera*]	1.00E-123	Fasciclin-like arabinogalactan protein
Contig5988	0.0099	0.01001	1.00908	_		No Hits Found
Contig2645	0.003	0.00292	0.97927	dbj|BAA22813.1| CND41, chloroplast nucleoid DNA binding protein [*Nicotiana tabacum*]	1.00E-166	Nucleoid DNA-binding protein cnd41-like protein
Contig3544	0.0033	0.00308	0.94748	emb|CAN68737.1| hypothetical protein [*Vitis vinifera*]	1.00E-107	7S globulin 2 precursor small subunit
Contig4994	0.007	0.00628	0.89978	emb|CAO65935.1| unnamed protein product [*Vitis vinifera*]	1.00E-94	Beta-adaptin
Contig1021	0.0089	0.00779	0.87871	gb|ABB13620.1| USP-like protein [*Astragalus sinicus*]	3.00E-58	Universal stress protein family protein
Contig1581	0.0184	0.01616	0.87812	emb|CAN65185.1| hypothetical protein [*Vitis vinifera*]	3.00E-57	Small heat-shock protein
Contig409	0.01	0.00857	0.8546	emb|CAO40936.1| unnamed protein product [*Vitis vinifera*]	7.00E-48	Bet v I allergen family protein/ PR10-like proteins
Contig1866	0.0102	0.00853	0.83941	emb|CAO40936.1| unnamed protein product [*Vitis vinifera*]	2.00E-49	Bet v I allergen family protein/ PR10-like proteins
Contig8158	0.0034	0.00282	0.82975	gb|ABK94910.1| unknown [*Populus trichocarpa*]	1.00E-111	E3 ubiquitin-protein ligase PRT1
Contig1010	0.0049	0.00405	0.8204	gb|AAP03998.1| EIL2 [*Nicotiana tabacum*]	0.00E+00	Ethylene-insensitive3-like1
Contig3075	0.007	0.00475	0.67981	emb|CAO15071.1| unnamed protein product [*Vitis vinifera*]	8.00E-38	Zinc finger (AN1-like) family protein
Contig3473	0.0082	0.0055	0.67422	gb|AAM63420.1| unknown [*Arabidopsis thaliana*]	4.00E-43	MD-2-related lipid recognition domain-containing protein
**Low KA/KS**						
Sequence	KS	KA	KA/KS	Firts Hit (BlastX-NR)	E-value	Annotation
Contig5300	0.0428	0.00179	0.04179	gb|ABK96261.1| unknown [*Populus trichocarpa x Populus deltoides*]	1.00E-125	Peroxisomal membrane protein-related
Contig3566	0.0238	0.00118	0.04963	emb|CAO24361.1| unnamed protein product [*Vitis vinifera*]	0.00E+00	Methionine aminopeptidase
Contig8165	0.017	0.00086	0.05052	gb|ABB87123.1| aspartic protease precursor-like [*Solanum tuberosum*]	9.00E-133	Aspartic proteinase
Contig4689	0.0509	0.00277	0.05441	gb|ABQ11264.1| mago nashi-like protein 1 [*Physalis pubescens*]	2.00E-76	Mago Nashi like protein
Contig8253	0.035	0.00205	0.05867	emb|CAO40052.1| unnamed protein product [*Vitis vinifera*]	6.00E-90	60S ribosomal protein L19
Contig6691	0.0181	0.00112	0.06173	dbj|BAA05641.1| chalcone synthase [*Camellia sinensis*]	0.00E+00	Chalcone synthase
Contig7328	0.024	0.00165	0.06904	emb|CAO61870.1| unnamed protein product [*Vitis vinifera*]	1.00E-62	Chloroplast photosystem II 22 kDa
Contig3171	0.0154	0.00114	0.07425	emb|CAI47559.1| alpha galactosidase [*Coffea arabica*]	0.00E+00	Alpha galactosidase
Contig8251	0.0125	0.00095	0.07596	gb|AAL99198.1| UTP:alpha-D-glucose-1-phosphate uridylyltransferase [*Solanum tuberosum*]	0.00E+00	UTP:alpha-D-glucose-1-phosphate uridylyltransferase
Contig3901	0.015	0.00115	0.07662	gb|ABF61806.1| alcohol dehydrogenase [*Dimocarpus longan*]	0.00E+00	Alcohol dehydrogenase
Contig944	0.0187	0.00144	0.07722	emb|CAO70082.1| unnamed protein product [*Vitis vinifera*]	8.00E-72	Nodulin MtN3 family protein
Contig8189	0.0477	0.00387	0.08126	dbj|BAA34348.1| elongation factor-1 alpha [*Nicotiana paniculata*]	0.00E+00	Elongation factor-1 alpha
Contig3388	0.0215	0.0018	0.084	emb|CAO70406.1| unnamed protein product [*Vitis vinifera*]	1.00E-37	Putative AP2/EREBP transcription factor

Contig4389	0.0238	0.00205	0.08607	gb|AAO85557.1| photosystem I subunit XI [*Nicotiana attenuata*]	4.00E-84	Photosystem I subunit XI precursor
Contig1320	0.0157	0.00147	0.09373	emb|CAO65178.1| unnamed protein product [*Vitis vinifera*]	2.00E-47	Zinc finger (C3HC4-type RING finger) family protein
Contig3416	0.024	0.00226	0.09405	gb|AAM18501.1| N-methyltransferase [*Coffea arabica*]	0	3,7-dimethylxanthine N-methyltransferase
Contig6826	0.0122	0.00117	0.09613	dbj|BAD34459.1| flavanone 3-hydroxylase [*Eustoma grandiflorum*]	0	Flavanone 3-hydroxylase
Contig2822	0.0087	0.00085	0.09744	gb|AAC61844.1| tyrosine/dopa decarboxylase [*Papaver somniferum*]	0	Tyrosine decarboxylase
Contig1066	0.0089	0.00089	0.09935	emb|CAN70603.1| hypothetical protein [*Vitis vinifera*]	0	Vacuolar-processing enzyme precursor (VPE)
Contig4399	0.017	0.00176	0.10343	emb|CAN79985.1| hypothetical protein [*Vitis vinifera*]	1.00E-113	Alpha-expansin precursor

ID: Contig number; KS: rate of synonymous substitutions, KA: rate of non-synonymous substitutions; KA/KS: KA to KS ratio; First Hit (BLASTX-NR): Most similar sequence in GenBank; E-value: E-value of most similar sequence; Annotation: automatic annotation based in AutoFACT results.
