# Supplementary material for: An EST-based analysis identifies new genes and reveals distinctive gene expression features of Coffea arabica and Coffea canephora
Source: BMC Plant Biol. 2011 Feb 8;11:30. doi: 10.1186/1471-2229-11-30 (PMC3045888; doi:10.1186/1471-2229-11-30)
Supplement: Additional file 5 — Top 20 Coffea spp. PFAM families. Word file containing the ranking of PFAM families in C. arabica and C. canephora. [file 1471-2229-11-30-S5.PDF]

## Additional File 5: Top 20 *Coffea* spp. PFAM families

| *Coffea arabica*[a] | Hits[b] | % Hits[c] | *Coffea canephora*[a] | Hits[b] | % Hits[c] |
|---|---|---|---|---|---|
| Pfam00069, Protein Serine/Threonine kinase | 235 | 2.38% | Pfam00069, Protein Serine/Threonine kinase | 127 | 2.32% |
| Pfam00067, Cytochrome P450 | 182 | 1.84% | Pfam00067, Cytochrome P450 | 72 | 1.31% |
| Pfam07714, Protein tyrosine kinase | 151 | 1.53% | Pfam07714, Protein tyrosine kinase | 68 | 1.24% |
| Pfam00076, RNA recognition motif | 102 | 1.03% | Pfam00076, RNA recognition motif | 60 | 1.10% |
| Pfam04554, Extensin-like region | 99 | 1.00% | Pfam03171, 2OG-Fe(II) oxygenase superfamily | 43 | 0.78% |
| Pfam07172, Glycine rich protein family | 82 | 0.83% | Pfam00071, Ras family | 39 | 0.71% |
| Pfam00106, Short chain dehydrogenase | 63 | 0.64% | Pfam00106, Short chain dehydrogenase | 38 | 0.69% |
| Pfam00153, Mitochondrial carrier protein | 59 | 0.60% | Pfam07172, Glycine rich protein family | 34 | 0.62% |
| Pfam00083, Sugar transporter | 58 | 0.59% | Pfam00005, ABC transporter | 33 | 0.60% |
| Pfam00179, Ubiquitin-conjugating enzyme | 58 | 0.59% | Pfam00153, Mitochondrial carrier protein | 31 | 0.57% |
| Pfam00201, UDP-glucoronosyl and UDP-glucosyl transferase | 53 | 0.54% | Pfam00450, Serine carboxypeptidase | 30 | 0.55% |
| Pfam00005, ABC transporter | 51 | 0.52% | Pfam00248, Aldo/keto reductase family | 28 | 0.51% |
| Pfam00226, DnaJ domain | 48 | 0.49% | Pfam04554, Extensin-like region | 28 | 0.51% |
| Pfam01370, NAD dependent epimerase/dehydratase | 48 | 0.49% | Pfam00004, AAA, atpase family | 28 | 0.51% |
| Pfam00450, Serine carboxypeptidase | 47 | 0.48% | Pfam00083, Sugar transporter | 25 | 0.46% |
| Pfam00931, NB-ARC domain | 45 | 0.46% | Pfam00481, Protein phosphatase 2C | 23 | 0.42% |
| Pfam00071, Ras family | 45 | 0.46% | Pfam00240, Ubiquitin family | 22 | 0.40% |
| Pfam03171, 2OG-Fe(II) oxygenase superfamily | 45 | 0.46% | Pfam00226, dnaj domain | 22 | 0.40% |
| Pfam00854, proton-dependent oligopeptide transport, POT | 44 | 0.45% | Pfam01490, Transmembrane amino acid transporter | 22 | 0.40% |
| Pfam02458, Transferase | 44 | 0.45% | Pfam00847, AP2 domain | 20 | 0.37% |
| Pfam00240, Ubiquitin family | 42 | 0.42% | Pfam00270, DEAD/DEAH box helicase | 20 | 0.37% |
| Pfam03552, Cellulose synthase | 42 | 0.42% | Pfam00190, Cupin | 20 | 0.37% |
| Pfam00004, AAA, ATPase family | 41 | 0.41% | Pfam00141, Peroxidase | 20 | 0.37% |
| Pfam00141, Peroxidase | 41 | 0.41% | Pfam00011, Hsp20/alpha crystallin family | 20 | 0.37% |
| Pfam00481, Protein phosphatase 2C | 40 | 0.40% | Pfam00504, Chlorophyll A-B binding protein | 20 | 0.37% |
| Pfam00248, Aldo/keto reductase family | 39 | 0.39% | Pfam02458, transferase | 19 | 0.35% |

a – PFAM family identity
b – Number of ESTS present in each family
c – Percenatge of ESTs present in each family
