# Supplementary material for: An EST-based analysis identifies new genes and reveals distinctive gene expression features of Coffea arabica and Coffea canephora
Source: BMC Plant Biol. 2011 Feb 8;11:30. doi: 10.1186/1471-2229-11-30 (PMC3045888; doi:10.1186/1471-2229-11-30)
Supplement: Additional file 6 — Annotation of Top 20 genes with the widest distribution among Coffea spp. cDNA libraries. Word file containing the ranking of genes distributed throughout coffee EST libraries. ID: Contig number; #: number of libraries represented in each contig; #ESTs: number of ESTs that compose each contig; First Hit (BLASTX-NR): Most similar sequence in GenBank; E-value: E-value of most similar sequence; Annotation: automatic annotation based in AutoFACT results. [file 1471-2229-11-30-S6.PDF]

# Additional File 6: Annotation of 20 genes with the widest distribution among *Coffea* spp. cDNA libraries

| ***Coffea arabica*** | | | | | |
|---|---|---|---|---|---|
| **Contig** | **#libraries** | **#ESTs** | **First Hit (BlastX-NR)** | **E-value** | **Annotation** |
| Contig1217 | 30 | 207 | gb\|EAZ38040.1\| hypothetical protein OsJ_021523 [*Oryza sativa* (japonica cultivar-group)] | 0 | Polyubiquitin |
| Contig9379 | 30 | 234 | gb\|ABK92924.1\| unknown [*Populus trichocarpa*] | 1.00E-165 | Cysteine proteinase |
| Contig16478 | 29 | 162 | gb\|ABK93203.1\| unknown [*Populus trichocarpa*] | 1.00E-163 | Glyceraldehyde 3-phosphate dehydrogenase |
| Contig16878 | 29 | 245 | gb\|AAY26520.1\| secretory peroxidase [*Catharanthus roseus*] | 1.00E-166 | Peroxidase |
| Contig3635 | 28 | 148 | emb\|CAO63006.1\| unnamed protein product [*Vitis vinifera*] | 8.00E-99 | Aquaporin 1 |
| Contig3702 | 28 | 147 | emb\|CAA66667.1\| polyubiquitin [*Pinus sylvestris*] | 0 | Polyubiquitin |
| Contig1691 | 27 | 203 | No hits Found | | |
| Contig3648 | 27 | 217 | sp\|P43396\|MT1_COFAR Metallothionein-like protein 1 (MT-1) | 3.00E-07 | Metallothionein |
| Contig1691 | 27 | 203 | No hits found | | |
| Contig4777 | 26 | 108 | gb\|ABK94573.1\| unknown [*Populus trichocarpa*] | 0 | eIF4-gamma/eIF5/eIF2-epsilon domain-containing protein |
| Contig3524 | 26 | 301 | emb\|CAA85426.1\| catalase [*Nicotiana plumbaginifolia*] | 0 | Catalase |
| Contig13370 | 25 | 194 | emb\|CAI56307.1\| sucrose synthase [*Coffea canephora*] | 0 | Sucrose synthase |
| Contig9414 | 25 | 81 | dbj\|BAA34348.1\| elongation factor-1 alpha [*Nicotiana paniculata*] | 0 | Elonagation Factor 1 |
| Contig6243 | 24 | 123 | emb\|CAN62488.1\| hypothetical protein [*Vitis vinifera*] | 0 | Heat shock protein 90 |
| Contig9342 | 24 | 79 | emb\|CAN69723.1\| hypothetical protein [*Vitis vinifera*] | 6.00E-85 | Eukaryotic translation initiation factor 5A |
| Contig16384 | 24 | 77 | gb\|ABF47216.1\| cathepsin B [*Nicotiana benthamiana*] | 1.00E-142 | Cathepsin B-like cysteine proteinase |
| Contig11332 | 24 | 107 | emb\|CAN81694.1\| hypothetical protein [*Vitis vinifera*] | 0 | Heat shock protein 70 |
| Contig2078 | 24 | 58 | gb\|AAQ63462.1\| calmodulin 8 [*Daucus carota*] | 4.00E-79 | Calmodulin |
| Contig1870 | 24 | 119 | emb\|CAN72774.1\| hypothetical protein [*Vitis vinifera*] | 1.00E-153 | YT521-B-like protein |
| Contig16384 | 24 | 77 | gb\|ABF47216.1\| cathepsin B [*Nicotiana benthamiana*] | 1.00E-142 | Cathepsin B-like cysteine proteinase |
| Contig9342 | 24 | 79 | emb\|CAO64503.1\| unnamed protein product [*Vitis vinifera*] | 6.00E-85 | Eukaryotic translation initiation factor 5A |
| Contig10847 | 24 | 111 | emb\|CAA58474.1\| methionine synthase [*Catharanthus roseus*] | 0 | Methionine synthase |
| Contig11332 | 24 | 107 | emb\|CAO21681.1\| unnamed protein product [*Vitis vinifera*] | 0 | Heat shock protein 70 |
| ***Coffea canephora*** | | | | | |
| **Contig** | **#libraries** | **#ESTs** | **First Hit (BlastX-NR)** | **E-value** | **Annotation** |
| Contig7932 | 9 | 22 | gb\|ABP65665.1\| VTC2-like protein [*Actinidia chinensis*] | 0 | GDP-l-galactose: hexose 1-phosphate guanylyltransferase |
| Contig559 | 8 | 87 | emb\|CAN72774.1\| hypothetical protein [*Vitis vinifera*] | 1.00E-148 | YTH2 protein; Pseudouridine synthase |
| Contig2001 | 8 | 66 | gb\|AAD03341.1\| ubiquitin [*Pisum sativum*] | 0 | Ubiqutin |
| Contig2882 | 8 | 31 | emb\|CAN74796.1\| hypothetical protein [*Vitis vinifera*] | 0 | Chaperonin |
| Contig3120 | 8 | 39 | gb\|AAC33305.1\| fiber annexin [*Gossypium hirsutum*] | 1.00E-128 | Annexin |
| Contig6320 | 8 | 63 | emb\|CAN73572.1\| hypothetical protein [*Vitis vinifera*] | 1.00E-139 | Single-stranded nucleic acid binding R3H |
| Contig6424 | 8 | 96 | gb\|ABK92924.1\| unknown [*Populus trichocarpa*] | 1,00 e-166 | Papain-like cysteine proteinase |
| Contig6667 | 8 | 30 | gb\|AAB39248.1\| NADP-isocitrate dehydrogenase [*Eucalyptus globulus*] | 0 | NADP-isocitrate dehydrogenase |
| Contig7234 | 8 | 25 | emb\|CAN68309.1\| hypothetical protein [*Vitis vinifera*] | 1.00E-94 | Tetraticopeptide domain-containing Thioredoxin |
| Contig8231 | 8 | 77 | emb\|CAI56307.1\| sucrose synthase [*Coffea canephora*] | 0 | Sucrose synthase |
| Contig5136 | 7 | 30 | emb\|CAO68932.1\| unnamed protein product [*Vitis vinifera*] | 1.00E-180 | Adenosine kinase isoform 2S |
| Contig3668 | 7 | 23 | gb\|AAA33697.1\| 1-aminocyclopropane-1-carboxylate oxidase [*Petunia x hybrida*] | 1.00E-140 | ACC oxidase |
| Contig1417 | 7 | 54 | emb\|CAC80550.1\| cyclophilin [*Ricinus communis*] | 3.00E-78 | Cyclophilin |
| Contig5950 | 7 | 23 | emb\|CAO17373.1\| unnamed protein product [*Vitis vinifera*] | 0 | Shaggy-related protein kinase alpha |
| Contig5037 | 7 | 51 | gb\|ABK95178.1\| unknown [*Populus trichocarpa*] | 1.00E-83 | Ubiquitin conjugating enzyme |
| Contig3525 | 7 | 30 | gb\|ABG33750.1\| cysteine protease [*Hevea brasiliensis*] | 0 | Cysteine proteinase |
| Contig811 | 7 | 47 | emb\|CAO69769.1\| unnamed protein product [*Vitis vinifera*] | 1.00E-154 | ADP, ATP carrier-like protein |
| Contig3198 | 7 | 42 | gb\|ABK94655.1\| unknown [*Populus trichocarpa*] | 1.00E-169 | Elongation factor 1 gamma-like protein |

| | | | | | |
|---|---|---|---|---|---|
| Contig6702 | 7 | 17 | dbj\|BAB68527.1\| 14-3-3 protein [*Nicotiana tabacum*] | 1.00E-128 | 14-3-3 protein |
| Contig4522 | **7** | 41 | gb\|EAZ23241.1\| hypothetical protein [*Oryza sativa*] | 0 | Translation elongation factor 2 |
| Contig1340 | 7 | 17 | emb\|CAO39543.1\| unnamed protein product [*Vitis vinifera*] | 1.00E-100 | pre-mRNA cleavage factor im, 25kD subunit |
| Contig978 | 7 | 26 | emb\|CAK22271.1\| 40S ribosomal protein S11 [*Chenopodium rubrum*] | 6.00E-74 | Ribosomal protein S11 |

ID: Contig number; #: number of libraries represented in each contig; #ESTs: number of ESTs that compose each contig; First Hit (BLASTX-NR): Most similar sequence in GenBank; E-value: E-value of most similar sequence; Annotation: automatic annotation based in AutoFACT results.
