# Supplementary material for: An EST-based analysis identifies new genes and reveals distinctive gene expression features of Coffea arabica and Coffea canephora
Source: BMC Plant Biol. 2011 Feb 8;11:30. doi: 10.1186/1471-2229-11-30 (PMC3045888; doi:10.1186/1471-2229-11-30)
Supplement: Additional file 7 — Annotation of 20 genes with the highest expression among Coffea spp. cDNA libraries. Word file containing the ranking of genes more expressed in coffee EST libraries. ID: Contig number; #: number of libraries represente in each contig; #ESTs: number of ESTs that compose each contig; First Hit (BLASTX-NR): Most similar sequence in GenBank; E-value: E-value of most similar sequence; Annotation: automatic annotation based in AutoFACT results. [file 1471-2229-11-30-S7.PDF]

Additional File 7: Annotation of 20 genes with the highest expression among *Coffea* spp. cDNA libraries.

Coffea arabica					
**Contig**	**#libraries**	**#ESTs**	**First Hit (BlastX-NR)**	**E-value**	**Annotation**
Contig16809	22	493	emb|CAD11991.1| rubisco small subunit [*Coffea arabica*]	5.00E-93	Rubisco small subunit
Contig5072	20	388	emb|CAO17297.1| unnamed protein product [*Vitis vinifera*]	1.00E-89	Sterol desaturase
Contig13384	15	383	No hits found		
Contig4415	17	376	emb|CAJ43737.1| class III chitinase [*Coffea arabica*]	7.00E-63	Class III chitinase
Contig1271	4	367	gb|AAK15088.1|AF240005_1 2S albumin [*Sesamum indicum*]	3.00E-09	2S albumin
Contig13751	18	333	dbj|BAA14339.1| cyc02 [*Catharanthus roseus*]	8.00E-14	Antimicrobial peptides precursor
Contig660	11	322	emb|CAJ43737.1| class III chitinase [*Coffea arabica*]	1.00E-120	Class III chitinase
Contig3524	26	301	emb|CAA85426.1| catalase [*Nicotiana plumbaginifolia*]	0	Catalase
Contig14309	23	273	gb|AAV44205.1| unknow protein [*Oryza sativa* (japonica cultivar-group)]	2.00E-25	Sucrose synthase
Contig2929	13	272	emb|CAA36249.1| metallothionein [*Mimulus guttatus*]	1.00E-17	Metallothionein
Contig16878	29	245	gb|AAY26520.1| secretory peroxidase [*Catharanthus roseus*]	1.00E-166	Peroxidase
Contig9379	30	234	gb|ABK92924.1| unknown [*Populus trichocarpa*]	1.00E-165	Hypothetical protein
Contig3648	27	217	sp|P43396|MT1_COFAR Metallothionein-like protein 1 (MT-1)	3.00E-07	Metallothionein
Contig1217	30	207	gb|AAD03341.1| ubiquitin [*Pisum sativum*]	0	Polyubiquitin
Contig16715	17	207	emb|CAN79558.1| hypothetical protein [*Vitis vinifera*]	1.00E-41	Major allergen Mal
Contig1691	27	203	gb|EES12155.1| hypothetical protein SORBIDRAFT_06g016540 [*Sorghum bicolor*]	1.00E-06	ABA/WDS induced protein
Contig16012	12	203	emb|CAA36249.1| metallothionein [*Mimulus guttatus*]	2.00E-18	Metallothionein
Contig12496	15	195	emb|CAA41188.1| chlorophyll a/b binding protein [*Nicotiana tabacum*]	1.00E-139	Chlorophyl a/b binding protein
Contig13370	25	194	emb|CAI56307.1| sucrose synthase [*Coffea canephora*]	0	Sucrose synthase
Contig15294	7	192	No hits found		
Coffea canephora					
**Contig**	**#libraries**	**#ESTs**	**First Hit (BlastX-NR)**	**E-value**	**Annotation**
Contig5887	6	1395	gb|AAK15088.1|AF240005_1 2S albumin [*Sesamum indicum*]	3.00E-08	2S albumin
Contig4069	5	725	gb|AAC61881.1| 11S storage globulin [*Coffea arabica*]	0	11S albumin
Contig2553	4	308	emb|CAO69959.1| unnamed protein product [*Vitis vinifera*]	2.00E-23	Lipid transfer protein
Contig2650	5	256	emb|CAJ40777.1| alpha galactosidase precursor [*Coffea arabica*]	1.00E-179	Alpha Galactosidase
Contig1953	6	216	No Hits Found		No Hits Found
Contig6917	5	212	ref|NP_190972.1| photoassimilate-responsive protein-related [*Arabidopsis thaliana*]	3.00E-34	PAR-1 protein
Contig3726	1	190	No Hits Found		No Hits Found
Contig2403	6	188	dbj|BAB90396.1| ADP-ribosylation factor [*Oryza sativa* (japonica cultivar-group)]	6.00E-99	ADP ribosylation factor
Contig2495	6	176	gb|AAY26520.1| secretory peroxidase [*Catharanthus roseus*]	1.00E-166	Peroxidase
Contig7356	6	173	emb|CAD11990.1| rubisco small subunit [*Coffea arabica*]	7.00E-85	Rubisco small subunit
Contig890	2	168	ref|NP_179721.1| mannose 6-phosphate reductase [*Arabidopsis thaliana*]	1.00E-118	Mannose 6-phosphate reductase
Contig2549	6	151	sp|P43396|MT1_COFAR Metallothionein-like protein 1 (MT-1)	2.00E-07	Metallothionein
Contig1103	5	150	emb|CAA95858.1| S-adenosyl-L-methionine synthetase 3 [*Catharanthus roseus*]	0	SAM synthase
Contig3776	4	150	ref|NP_566847.1| unknown protein [*Arabidopsis thaliana*]	2.00E-86	Hypothetical protein
Contig3742	4	132	emb|CAJ43737.1| class III chitinase [*Coffea arabica*]	5.00E-64	Class III chitinase
Contig4591	2	128	emb|CAO49414.1| unnamed protein product [*Vitis vinifera*]	1.00E-106	WRKY family transcription factor
Contig3494	5	127	emb|CAA66109.3| specific tissue protein 2 [*Cicer arietinum*]	7.00E-05	Hypothetical protein
Contig6863	7	126	gb|ABB29942.1| S-adenosyl methionine synthase-like [*Solanum tuberosum*]	0	SAM synthase
Contig7290	6	124	emb|CAA85426.1| catalase [*Nicotiana plumbaginifolia*]	0.00E+00	Catalase
Contig6466	4	118	gb|ABK92757.1| unknown [*Populus trichocarpa*]	1.00E-117	Mob1-like protein

ID: Contig number; #: number of libraries represented in each contig; #ESTs: number of ESTs that compose each contig; First Hit (BLASTX-NR): Most similar sequence in GenBank; E-value: E-value of most similar sequence; Annotation: automatic annotation based in AutoFACT results.
