# Supplementary material for: An EST-based analysis identifies new genes and reveals distinctive gene expression features of Coffea arabica and Coffea canephora
Source: BMC Plant Biol. 2011 Feb 8;11:30. doi: 10.1186/1471-2229-11-30 (PMC3045888; doi:10.1186/1471-2229-11-30)
Supplement: Additional file 9 — Results concerning some genes related with drought stress (Dehydrins, LEAs and Metallothioneins). Word file describing results and a brief discussion about dehydrins, LEAs and Metallothioneins expressed in coffee EST libraries. [file 1471-2229-11-30-S9.PDF]

Additional File 9: Results concerning some genes related with drought abiotic stress (Dehydrins, LEAs, Metallothioneins).

Dehydrins are extensively characterized as proteins expressed during drought stress [1]. However, Hinniger et al. [2] isolated and characterized dehydrins expressed during *C. canephora* and *C. arabica* fruit development. For both species they found that dehydrin CcDH2 and CcDH1 are expressed during the final stages of grain development, but CcDH1 are also detected in the pericarp, leaves and flowers. CcContig7329 corresponds to CcDH1a isoform and, according to expression clustering analysis, it seems to be preferentially expressed in leaves (Additional File 7). CcContig1448, which corresponds to CcDH2, is mostly expressed in SE3 library (Middle stage seeds; Additional File 7), coinciding with previous data [2]. Other desiccation tolerance-related gene characterized by those authors was a *LEA* (Late Embryogenesis Abundant) [3] detected during a brief period of mid-stage development. CcContigs 1491 and 7919 were also preferentially expressed in SE3 library (Additional File 7; Figure 6A).

Metallothioneins (MTs) are small Cys-rich proteins that bind essential and non-essential heavy metals. MTs are involved in zinc (Zn) homeostasis and have antioxidant function [4,5]. There is evidence that MTs scavenge oxygen free radicals and avoid DNA damage and lipid peroxidation [4]. In *C. arabica* 6 MTs were found to be preferentially expressed in libraries from plants treated with arachidonic acid (AA) (Additional File 7). AA is a polyunsaturated fatty acid (PUFA) present in pathogens, such as oomycete *Phytophthora* spp. AA has toxic effects, which are associated with mitocondrial damage and lipid peroxidation that can induce program cell death in plants [6]. It was suggested that zinc has a protective role against AA toxicity by inducing MT that could scavenge ROS, alleviating the stress [7]. In this scenario, the amount of MTs

expressed in plants treated with AA can be a consequence of a protective signaling cascade against damaging effects of such substance.

REFERENCES

1. Allagulova Ch R, Gimalov FR, Shakirova FM, Vakhitov VA: **The plant dehydrins: structure and putative functions**. *Biochemistry (Mosc)* 2003, **68**(9):945-951.

2. Hinniger C, Caillet V, Michoux F, Ben Amor M, Tanksley S, Lin C, McCarthy J: **Isolation and characterization of cDNA encoding three dehydrins expressed during *Coffea canephora* (Robusta) grain development**. *Ann Bot* 2006, **97**(5):755-765.

3. Wise MJ, Tunnacliffe A: **POPP the question: what do LEA proteins do?** *Trends Plant Sci* 2004, **9**(1):13-17.

4. Bourdineaud JP, Baudrimont M, Gonzalez P, Moreau JL: **Challenging the model for induction of metallothionein gene expression**. *Biochimie* 2006, **88**(11):1787-1792.

5. Freisinger E: **Plant MTs-long neglected members of the metallothionein superfamily**. *Dalton Trans* 2008(47):6663-6675.

6. Knight VI, Wang H, Lincoln JE, Lulai EC, Gilchrist DG, Bostock RM: **Hydroperoxides of fatty acids induce programmed cell death in tomato protoplasts**. *Physiol Mol Plant Pathol* 2001, **59**(6):277-286.

7. Perez MJ, Cederbaum AI: **Metallothionein 2A induction by zinc protects HEPG2 cells against CYP2E1-dependent toxicity**. *Free Radic Biol Med* 2003, **34**(4):443-455.
