# Supplementary material for: An EST-based analysis identifies new genes and reveals distinctive gene expression features of Coffea arabica and Coffea canephora
Source: BMC Plant Biol. 2011 Feb 8;11:30. doi: 10.1186/1471-2229-11-30 (PMC3045888; doi:10.1186/1471-2229-11-30)
Supplement: Additional file 10 — RALF and RALF-like peptides. Word file containing the sequences of RALF and RALF-like peptides expressed in coffee. In magenta: dibasic sites; in yellow: cysteine residues. [file 1471-2229-11-30-S10.PDF]

Additional File 10: RALF and RALF-like peptides in *Coffea* spp. In magenta: dibasic sites; In Yellow: cysteine residues

A) RALF Peptides

Coffea arabica

CaContig13668
MGWMMMPGMARSGLVGEDDGVEFELDSESNRRILATTRYISYGALQKNSVPCSRRGQSYYNCRPGAPANP
YSRGCSAITRCRS

CaContig4015
MLVSFWAVGDAASGSHELSYYFPAVTTSTASFCNGGSIESCLMSEQEEELEMDSETNRRILYWRRRYISY
SALTRDRVPCSRRGYSYYNCRPGRPVNPYNRGCNAITRCRR

Coffea canephora

Contig3558
MANSSSLSTLLFALSLLTALVLSSTVVSASGGDHYDAAQMGWMMMPGMARSGLVGEDDGVEFELDSESNR
RILATTRYISYGALQRNSVPCSRRGQSYYNCRPGAPANPYTRGCSAITRCRS

Contig742
MVKPSAGLFLISATLFAATMLVSFWAVGVAASGSHELSYYFPAVTTSTASFCNGGSIESCLMSEQEEEGD
DDDQEELEMDSETNRRILYWRRRYISYGALTRDRVPCSRRGYSYYNCRPGRPVNPYNRGCNAITRCRR

Contig4772
MAKSGLAGKNNGGEFKLDSKSNGGILATTRYIS*GALQRNRAPCSRRGKFYYNCRPGAPVNPYT
RGCRAITRCRSKNFRTSIHLAKSFGFPFPFPLGGK

CC00-XX-PP1-077-C04-TL.F
MSVLDLNSMKNGELDAMVKRACAGKMSDCPTVSLEEEEEEMDSESHRRMLLMRRRFISYDTLRRDFAPCN
RPGSSYYNCKGAGPVNTYNRGCEIITRCDRGD

Contig3823
MMSLYDAADDVVVDNDDEMEMDDDAVSSSRRSLFWRRVRYYISYAALSANRIPCPPRSGRSYYTHHCYFA
SGPVHPYNRGCSAITRCRR

B) RALF-like Peptides

Coffea arabica

CaContig203

MEKSASKSLCIFPVVAWLQLTTTVLLSATSIQSVNASVSWDWGDSTVGSTVVADDQEFLMDSQFGNVLAS
GGSNVYRALGRKPICNNARYANCLGAGANGRPCDYTNRCAKH

CA00-XX-RM1-050-F02-AC.F

MEKSASKSLCIFPVVAWLLLITTVLLSATSIQSVNASVSWDWDNSTVGSTVADDQEFLVDSQFGNVLAVP
QQRYVTRRVLQPPPICDRTRYANCIQPGANQRPCDLHNRCARHI

Coffea canephora

CcContig7693

WSPSKSKSLCIFPAVAWLLLTTIVLLSATSIQSVNGSISWDWGNSTIGSTTAGDQEFLMDSQFGNVLAS
RRGVAYRVLGRKPICNNPRYANCIGAGANGRNCGYDNRCLRHS

CcContig15

MEKSASKSLCIFPVVAWLLLTTTLLLSATSIQSVNGSVGWDWGDSTVGSTVVADDQEFLMDSQFGNVLAS
GVSTSKVPLQKGPFCSRLYYNHCIQRFGRDPKDRECDYTNHCGRQSPH

CcContig5266

KSKSKSKSKSLCIFPVVAWLLLTTTLLLSATSIQSVNGSVSWDWGDPTVGSTVVVDDQEFLVDSQFGNVL
AVPPRGKSLSYRGLEQPAICGLAVYYHCIQRFGRDPKDRECLYRELCRH

Contig2070

MEKSAPKSLCIFPVVAWLLLTTTVLLSAASIQSVNGSVSWDWDNSTVGSTVADDQEFLMDSQFGNVLASG
GSNVYRALQRKPFCDNARYANCIGAGAKANGSPCRFSDHCRHNVG

CC00-XX-LF1-040-H01-TL.F
MEKSAPKSLCIFPVVAWLLLTTTVLLSAASIQSVNGSVSWDWDNSTVGSTVADDQEFLMDSQFGNVLASG
GSNVYRALQRKPFCDNARYANCIGAGAKANGSPCRFSDHCRHNVG
