# Supplementary material for: An EST-based analysis identifies new genes and reveals distinctive gene expression features of Coffea arabica and Coffea canephora
Source: BMC Plant Biol. 2011 Feb 8;11:30. doi: 10.1186/1471-2229-11-30 (PMC3045888; doi:10.1186/1471-2229-11-30)
Supplement: Additional file 11 — OrthoMCL families of Glycine Rich Proteins (GRP). Word file containing the sequences of Glycine Rich Proteins expressed in coffee. In yellow: cysteine residues; Underlined: signal peptide for secretion. [file 1471-2229-11-30-S11.PDF]

Additional File 11: *Coffea* spp. OrthoMCL families of Glycine Rich Proteins (GRP). In Yellow: cysteine residues; Underlined: signal peptide for secretion

A) Family1231 – Class I-like GRPs

Coffea arabica

CA00-XX-CB1-036-E04-MC.F
MAVVLMITSEVAAKSVDNSKTVETNEEGEAKYHGGGYGGGHGGGYGGGHGGHGGGYGGGHGGGHGGHHGG
GYGGHPGEGNGDGHGGYGGGGHGGYGHGGGSHGGYGHGGHGGGGHGGHPGEAADAKPQN

CaContig13520
MGSKTLLFFFISMAVVLMITSEVAAKSVDNSKTVETNEEGEAKYHGGGHGGGHGGGYGGGHGGHGGGYGG
GHGGGHGGHHGGGYGGHPGEGNGDGHGGYGGGGHGGYGHGGGGHGGYGHGGHGGGGHGGHPGEAADAKPL
N

CA00-XX-CS1-066-A03-CC.F
ISLHFHGFQDTSFLFHFPGCSSNDHLRGGYGGGHGGGHGGGYGGGHGGGHGGHHGGGYGGHPGGGNGDGH
GGYGGGGHGGYGHGGGGHGGYGHGGHGGGGHGGHPGEAADAEPQN

CaContig11073
IRYHTQFTSISHHHGGGHGGGYGGGHGGHGGGYGGGHGGGHGGHHGGGYGGHPGEGNGDGHGGYGGGGHG
GYGHGGGGHGGYGHGGHGGGGHGGHPGEAADAKPLN

CaContig13384
MGSKTLLFFFISLAVVLMITSEVAAKSVDNSKTVETNKEGEAKYHGGGYGGGHGGGHGGGYGGGHGGGHG
GHHGGGYGGHPGGGNGDGHGGYGGGGHGGYGHGGGGHGGYGHGGHGGGGHGGHPGEAADAEPQN

CaContig14011
MGSKTLLFFFISMAVVLMITSEVAAKSVDNSKTVETKEEGEAKYHGGGYGGHHGGGYGGGHGGYGGGGHG
GHPGEAADAEPQ

CaContig16626
MGSKTLLFFFISMAVVLMITSEVAAKSVDNSKTVETNEEGEAKYYGGGHGGGHGGGHGGGYGGGHGGGHG
GHHGGGYGGHPGGGYGGGHGGYGGGGHGGYGHGGHHGGGGHGGHPGEAADAQPQN

CaContig5866 (?)
MSSMKILFFCISLALVLMITSQVAARELVEITSNSVDNSKTDEANGLKEAKYPGGYGGYPGGGYGGYPGG
GYGGYPGGGYEGYPGGGYGGYPGGRYGGYPGGRYGGYPGGGRGGYGGNCRFGCCGRNYYGGGCRCCYYPG
QAVDAEPQN

CaContig14011
MGSKTLLFFFISMAVVLMITSEVAAKSVDNSKTVETKEEGEAKYHGGGYGGHHGGGYGGGHGGYGGGGHG
GHPGEAADAEPQN

CaContig16172
MGSKTLLFFFISMAVVLMITSEVAAKSVDNSKTVETNEEGEAKYHGGGYGGGHGGGHGGGYGGGHGGGYG
GHHGGGYGGGHGGYGGGGHGGYGHGGHHGGGGHGGHPGQAAGAEPQN

CaContig8936
MGSKTLLFFFISMAVVLMITSEVAAKSVDNSKTVETNEEGEAKYHGGGYGGGHGGGYGGGHGGGYGGHHG
GGYGGGHGGYGGGGHGGYGHGGHHGGGGHGGHPGEAADAEPQN

CA00_XX_CB1_108_B01_RF_F
MGSKTLLFFFISMAVVLMITSEVAAKSVDNSKTVETNEEGEAKYLTVTTHLTSRHTMFPIHQSCHQFTRS
HHLMVTTHQFTRSHQFTRSHHLMVTTHLTSRHIMFPSHQSCHQYTRSHHLMVTTHLTSRHGGHGGGYGGG
HGGGHGGHHGGGYGGHPGEGNGDGHGGYGGGGHGGYGHGGGSHGGYGHGGHGGGGHGGHPGEAADAKPQN

CA00_XX_PA1_013_C02_EC_F
MGSKTLLFFFISMAVVLMITSEVAAKSVDNSKTVETNEEGEAKYHGGGGHGGYGHGGHGGGGHGGHPGEA
ADAKPLN

Coffea canephora

CC00-XX-SH3-001-A09-EM.F
SHVWYGGGGHGGYGHGGGGHGGYGPADYAHGRHGGGGHGGHPGEAADAHPQT

CcContig2017
MGSKTLLFFFISMAVVLMITSEVAAKSVDNSKTVETNEEGEAKYHGGGYGGGHGGGYGGHGGGYGGGHGG
HHGGGYGGHPGEGNGDRHGGYGGGGHGGYGHGGHGGGGHGGHPGEAANAKPQN

CcContig376
MGSKTLLFFFISMAVVLMITSEVAAKSVDNSKTVETNEEGEAKYHGGGYGGGHGGGYGGHGGGYGGGHGG
HHGGGYGGHPGEGNGDRHGGYGGGGHGGYGHGGGGHGGYGHGGHGGGGHGGHPGEAADAKPQN

CcContig1840
MGSKTLLFFFISMAVVLMITSEVAAKSVDNSKTVETNEEGEAKYHGGGYGGGHGGGYGGHGGGYGGGHGG
HHGGGYGGHPGEGNGDRHGGYGGGGHGGYGHGGGGHGGYGHGGGGHGGYGHGGHGGGGHGGHPGEAADAK
PQN

CcContig6984
MGSKTLLFFCISLAIVLTIASQVAARELAETTTSAENSKTDETTGVEEGKYGGGYGGYGGYPGYGGYGGR
GGYGGYGGRGGYGGYGGRGGYGGYGGRGGYGGYGGRGGYGGYPGGGYGGRGGYGGYPGGGYGHGGYPGQA
VDAEPQN

B) Family4011 - Class II-like GRPs

Coffea arabica

CA00-XX-CA1-004-H01-EZ.F
MGSKAILLLCLLAAVLMIASEVTARDLAENTNAAEKSTEGLEESKYYGGGGRYGGGGGHYGGGGGHYGGG
GGHYGGGGGHYGGGGGGCYHGCCGGGGYGGCRCCTYAGEPKDAGYTEPETKPQ

CaContig5329
MGSKAILLLCLLAAVLMIASEVTARDLAENTNAAEKSTEGLEESKYGGGGGRYGGGGGRYGGGGHCYGGH
CGGGGGGGHYGGGGGGCNHGCCGGGGYGGCRCCTYAGEPKDAGYTEPETKPQN

CaContig6646
MSSKAILLLCLLAAVLMIASEVTARDLAENTNAAEKSTEGLEESKYGGGGGCHGYGCGGGGGGCHGYGCH
GGGGGGGGGHCYHGCCGHGYGGCRCCTYAGEPKDAGYTEPETKPQN

CaContig2625
MGSKAILLLCLLAAVLMIASEVTARDLAENTNAAEKSTEGLEESKYGRGGGCYGRGCGGGGGGGRCYHGC
CGGGYGGCRCCTYAGEPKDAGYTEPETKPXN

CA00-XX-LP1-021-G09-EB.F
MGSKAILLLCLLAAVLMIASEVTARDLAENTNAAEKSTEGLEESKYGGGGGCHGYGCGGGGGGHCYGGHC
GGGGGGGHCYHGCCGHGYGGCRCCTYAGEPKDAGYTEPETKPQ

CaContig16496
MGSKAILLLCLLAAVLMIASEVTARDLAENTNAAEKSTEGLEESKYYGGGGGGGRGCYGRGCGGGGGGCY
HGCCGGGGGYGYGHGGCRCCTYAGEPKDAGYTEPETKPQN

CaContig1435
MGSKAILLLCLLAAVLMIASEVTARDLAENTNAAEKSTEGLEESKYGRGGGCYGRGCGGGGGGGCHGYGC
GGGGGGGGHGCYHGCCGGGYGGCRCCTYAGEPKDAGYTEPETEPQN

CaContig3765
MGSKAILLLCLLAAVLMIASEVTARDLAENTNAAEKSTEGLEESKYYGGGGHYGGGGGHYGGGGGHYGGG
GGHYGGGGGHYGGGGGHYGGGGGCYNGCCGHGYGGCRCCTYAGEPKDAGYTEPETKPQN

CA00-XX-IA2-030-G11-EC.F
LAENTNAGEKSNEGLEESKYGGGGGCHGYGCGGGGGGHHCYHGCCGHGYGGCRCCTYAGEPKDAGYTEPE
TKPQN

CA00-XX-LP1-002-E03-EB.F
MGSKAILLLCLLAAVLMIASEVTARDLAENTNAAEKSTEGLEESKYGRGGGCHGYGCGGGGGGWWPWLLP
WLLWRRLWRLQMLHICW

C) GRPs differentially expressed in *C. arabica* plantlets treated with AA containing 12 Cys

CaContig10126
MGSKAILLLCLLAAVLMIASEVTARDLAENTNAAEKSTEGLEESKYGRGGGCYGRGCGGGGGGCYGGHCG
GGGGGGHCYGGHCGGGGGGGHGCYHGCCGGGYGGCRCCTYAGEPKDAGYTEPETKPQN

CaContig1089
MGSKAILLLCLLAAVLMIASEVTARDLAENTNAAEKSTEGLEESKYGGGGGCHGYGCGGGGGGCHGYGCG
GGGGGHCYGGHCGGGGGGGHCYHGCCGHGYGGCRCCTYAGEPKDAGYTEPETKPQ

CaContig3317
MGSKAIFLLCLLAAVLMIASEVTARDLAENTNAAEKSTEGLEESKYGRGGGGCYGGHCGGGGGCYGGHCG
GGGGGGHCYGGHCGGGGGHGCYHGCCGGGGYGGGCRCCTYAGEPKDAGYTEPETKPQN
