# Supplementary material for: An EST-based analysis identifies new genes and reveals distinctive gene expression features of Coffea arabica and Coffea canephora
Source: BMC Plant Biol. 2011 Feb 8;11:30. doi: 10.1186/1471-2229-11-30 (PMC3045888; doi:10.1186/1471-2229-11-30)
Supplement: Additional file 12 — OrthoMCL families of Cystatins. Word file containing the sequences of Cystatins expressed in coffee. In green: variation of LARFAV motif; in yellow: new motif GG-X-YY; in blue: QVVAG motif. [file 1471-2229-11-30-S12.PDF]

Additional File 12: *Coffea* spp. OrthoMCL families of cystatins: In green: variation of LARFAV motif; In yellow: new motif GG-X-YY; In blue: QVVAG motif

Family544

Coffea arabica

CA00_XX_AR1_001_B01_EB_F
MAAAKFAIGTGQTDISSLEPVKPADPHVIQIGKFVEQQHHGKLLSVAVVGGFTWSGDG
GNYYALIIENQDSDGATCLHKHKVLVLETPSETKLIWHKK

CaContig113
MSTVAARSATPAIGAGQKNMVGVPCVPMASTVKRTDPGVIGIANFAVEKYNERNETALA
VINVEFGFLWPHGGHYYYMLAIITQDDKGTHHDVAYVRDAGKSNAHAYEFMWYNHNNN

CA00_XX_LP1_007_B05_EB_F
MDQVPVNPEDVDRIGRFAVNEENRKRPNQLTFVHVVYAFKGSAGEDKIYPLIIKIRDVN
DKPFKHKALVLEKTDGSLNLKGYWE

CA00_XX_RT8_064_F07_EQ_F
MAAAKSAIGTGKNDISSLEPVKPADRHVIQIGEFVVEQCHHGQLLFVAVVGGFTWSGDG
GYYYALIIENQDSEGATYLHKALVLETPNETKLIWHKK

CaContig8767
MAAAKSAIGTGKNDISELEPVKPADPHVVQIGEFVVEQCHHGQLLFVAVVGGFTWSGDG
GYYYALIIENQDSDGATYLHKALVLQTPSETKLIWHKK

CaContig10690
MAAAKSAIDTGKNDISSLEPVKPADPHVIQVGQFVVEQCYHGQLLFVAVVGGFTWSGDG
GYYYALIIENQDNDGATYLHKALVLETPSETKLIWHKK

CA00_XX_RX1_054_H06_EB_F
MPGQIDVDGLVPVKPTDPPVIAIGKFAVEEYKKKQPIEIVAVVSGFTGSGDGGNYYLLI
IETQDSNGAIFLHKALVFKDTNGGLKVKGYWGF

CaContig13279
MAAVAANFPVAGVAKNPMQGLKPALVVGALNQLAGQKQGQGNAAVPDDWTPVNPLDRHI
QELGAFAVDEHNKQTKDQLVFVAVLSGIQKTEDDRSTYCLLISAKDSTGKLGRYYAVII
EYNTGCQQLLQFEPSP

CaContig11025
MAAAKSAIGTGQTDISSLEPVKPADPHVIQIGRFVVEQAHHGKLLFVAVVGGFTWSVIG
GNYYALIIENQDYEGATYLHKALVFETPDGVLELIWHKK

CaContig5403
MAAAKSAIGTGQTDISSLEPVKPADPHVIQIGQFVVEQAHHGKLLFVAVVGGFTWSVIG
GNYYALIIENQDYEGATYLHKALVFETPDGVLTLIWHKK

CaContig16944
MAEAKSATVTDQIDINSIQPVAPADPHVVGIGQFVVEKFHHGKLLFIAVLGGFTWKCEG
GKYYALIIQNQDYEGATFIHKALVVEAKGETKLLWHRN

CaContig4522
MAEAKSATVTDQIDITSIQPVAPADPHVVGIGQFVVEKFHHGKLHFIAVIGGFTWNCEG
GKYYALIIQNQDYEGATFIHKALVVEAKGETKLLWHRN

CaContig16895
MAAAKSAIGTGQTDISSLEPVKPADPRVIQIGQFVVEQAHHGKLLFVAVVGGFTWSVIG
GNYYALIIENQDYEGATYLHKALVFETPDGVLTLIWHKK

CaContig8410
MAEAKSATVTDQIDINSIQPVKPADPRVVEIGQFVVEKFHHGKLLFIAVLGGFTWKCEG
GKYYALVIENQDYEGATFIHKALVVEAPGETKLLWHKN

CaContig4566
MAAAKSAIGTGQTDISSLEPVKPADPHVIQIGQFVVEQAHHGKLLFVAVVGGFTWSVIG
GNYYALIIENQDYEGATYLHKALVFETPDGVLTLIWHKK

CaContig12045
MAKFSVDKYNEEAGTKLVFMKVIACALWNLGVVTVYALLIQTQDSKGTYIDKAVAVDVT
IIGKKLLWYKH

CA00-XX-RT8-047-A07-EP.F
MAAAKSAIGTGKNDISSLEPVKPADPHVIQIGQFVVEQCHHGQLLFVAVVGGFTWSGDG
GYYYALIIENQDSDGATYLHKALVLETPSETKLIWHMK

CaContig15921
MVGVPCVPMASTVKRTDPGVIGIANFAVEKYNERNETALAVINVEFGFLWPHGGHYYYM
LAIITXDDKGTHHDVAYVRDAGKTMLTLMNSCGTIITIIDLALLLIS

CaContig8137
MAAAKSAIGAGKNDIDALEPVKPADPRVIEIGRFAVTEHGHALLFVGVVGGFRWAIPGG
DHYALIIETQDDNGATYLHKALVVMVEVEGQPLRLIWYKN

CaContig17257
MAAAKSAIGTGKIDISSLEPVKPADPHVIQIGKFVEQQHHHGKLLCVAVVGGFTWSGDG
GNYYALIIENQDSDGATYLHKHKVLVLETPSEMKLIWHKK

Coffea canephora

CcContig2160

MAEAKSATVTDQIDINSIQPVAPADPHVVGIGQFVVEKFHHGKLLFIAVLGGFTWKCEG
GKYYALIIQNQDYEGATFIHKALVVEAKGETKLLWHRN

CcContig4504

MATVAAKSATAAIGAGQKNMVGGGLSSTVPPRSSTVNPKDPHVIQIAQFAVANYNAKAG
TTVVWLNVEYGFWWIDDDTYYMLAIKTQDLTGTHCDVALVREISESNGTYSLKWYNHNN
K

CC00-XX-PP1-087-G12-TL.F

MDQVPVNPEDVDRIGRFAVNEENRKRPNQLTFVHVVYAFKGSAGEDKIYPLIIKIRDVN
DKPFKHKALVLEKTDGSLNLKGYW

CcContig3825

MSTVAARSATPAIGAGQKNMMGGGVSCIIPPATTVKVEDACVIEIAKFAVAQITGRVFI
KVEFGFWWKIEIGPNAGTYYMLAIITQDNNRTHCDVALVCDLETSNGHTLIWYNDKNN

CcContig7886

MAEAKSATVTDQIDINSIQPVAPADPRVAEIGQFVVEKFHHGKLLFIAVIGGFTWKCEG
GKYYALIIQNQDYEGATFIHKALVVEAPGETKLLWHRN

CcContig1043

MVGGGLSSTVPPRSSTVNPKDPHVIQIAQFAVANYNAKAGTTVVWLNVEYGFWWIDDDT
YYMLAIKTQDLTGTHCDVALVREISESNGTYSLKWYNHNNK

Family2703

Coffea arabica

CaContig1058
MTEVIANYNISVNEFAANMAVEGFQSAEVEAIMKAVGENKTWNAIEGLSDTNANLRGLC
GTTTAQNVDKTVPPDVQEMAEFAVAEYNRIAGTKLVLIKVLAYVKLVVVFGTFYGLHML
TQDDKGTYKDQALTLKLNNGMKVLLWYKHN

CaContig4053
MAVTAKCQKTELANNYVKQFQSAEVDAILKQAGETKLIVHGGWTPVNPADPHIQELGRF
AVDEHNKQTGDKLVFVAVVAGLKKPVELATLYWLIIEAKDSDGNQNIYKALVQETDLEM
KKLLYFGEVVPPVN

CaContig5345
MTEVIANYNINVNEFAANMAVEGFQSAEVEAIMKAVGENKTWNAIEGLSDTNANLQGLC
GTTTAQNVDKTVPPDVQEMAEFAVAEYNRIAGTNLVLIKVLAYVKRVVVFGTLYRLHML
TQDDKGIHNDQALTLKLKNGKKVLLSYKHN

CaContig4160
MAAAKSGIGSGQKDEPIIPMASTVNPNDDVVIQKAKFAVDSYNGQAGTGLKFNSVEFGF
CWSVSDVTDYLLAINTHDDKGPYCDPALVSDTLKSNAHTYELIWYNHKKK

CaContig7667
MATVAAKSATAAIGAGQKNMVGGGLSSTVPPRSSTVNPKDPHVIQIAQFAVANYNAKAG
TTVVWLNVEYGFWWIDDDTYYMLAIKTRDLTGTHCDVALVREISESNGTYSLKWYNHNN

CaContig7242
DPHIQELGRFAVNEHNRQTRDKLVFVAVVAGLKKPVELATLYWLIIEAKDRNGNQNIYK
A

Coffea canephora

CcContig6730
MTEVVANYNINVNEFAANMAVEGFQSAEVEAIMKAVGENKTWNAIEGLSDTNANLQGLR
GTTTAQNVDKTVPPDVQEMAEFAVAEYNRIAGTNLVLIKVLAYVKRVVVFGTLYGLHML
TQDDKGIHHDQALTLKFKNGKKVLLWYKHNKH

CcContig4176
MTEVIANYNINVNEFAANMAVEGFQSTEVEAIMKAVGENKTWNAIEGLNDTNANLQGLC
GTTTAQNVDKAVPPDVQEMAEFAVAEYNRRAGTKLVLIKVLRYVKRVVVFGTFYGLHML
TQDDKGTYKDQALALKFKNGKKVLVWYKHNEN

CC00-XX-SH3-075-F10-EM.F
MAAAKSGIGSGQKDQPIIPVASTVKPKDDKVIEAAQFAVVTYNKQAGTDLVCINVEFGF
WWSITGATYYMLAIKTQDAKGTYCHVALVADVLVSGGNHTYDLIWYNHKN

Family942

Coffea arabica

CaContig2092
MAAVVANPHINITEITANMKAEGVQSPEIEAIVKALSDDTIWKTIEGFKGKDMSTQEKM
INNMVAGGHLPQVGVPLPTPVNPTDPHVISVAKFALAKYNDKHGTKLVFNRVNGGLQWK
IVIGTLYILVLATQDSKGTYTDYAVVFETFLGQKYLFWYKH

CaContig2323
MAAVAANFPVAGVAKNPMQGLKPALVVGALKQLAGQKQGQGNAAVPDDWTVVSTLDRHI
QALGAFAVDEHNKQTKNQLVFVAVLSGIKKTEDDRSTYCLLISAKNSTGKLGSYNAVII
EYNTGCQQLLQFEESP

CaContig 13328
MDLVPVNPAEPHVTAIGQFAVDEENKKRPTNKLNFVAVVGGYHGPVTGATRYPLILGTQ
DGKGHTFLHKALVHEKPDGSLELKGYW

CaContig5297
MASAFPHLLLLTTLAAICLFSDVPSAALGGRPKDALVGGWSKADPKDPEVVENGKFAVD
EHNKEAKTKLEFKTVVEAQQQVVAGTNYKIVIKALDGTASNLYEAIVWVKPWLKFKKLT
SFRKLP

CaContig15270
MASAFPHLLLLTTLAAICLFSDVPSAALGGRPKDALVGGWSKADPKDPEVVENGKFAID
EHNKEAGTKLEFKTVVEAQEQVVAGTNYKIVIKALDGTASNLYEAIVWVKPWLKFKKLT
SFRKLP

CaContig14147
MDMCDEDFFVTGGGKDTKLVGIAGVPLPKPVDKTSPHVIKIAQFAVKKHNEKAGTKLVF
IKVVGGVKWSAIAGTFYALQIETQDSKGTYRDKTLVVEAVTGHKKLIWYKH

CaContig3848
MTEVTVNYNFNITEVAANMAVEGFQSAEVEAIMKTAGDDMIWNAIEDTKDMDMCDEDFF
VTGGGKDTKLVGIAGVPLPKPVDKTSPHVIKIAQFAVKKHNEKAGTKLVFIKVVGGVKW
SAIAGTFYALQIETQDSKGTHRDKTLVVEAITGHKKLIWYKH

CA00-XX-IA2-005-D11-EC.F
MAAVVANYNINISEITANMKAEGVQSPEMEAILKATAEDAIWNTIERFKGMDMSNKKKM
INNRMGSGGRAQLGIPLPEPVNPTDPHVIAIAKFAVEKHNENAGTSLVFIQVIGGLQWN
LLIGALYMLIITTQDSKGTYYDKTVVFETCLGQKYLLWYKH

Coffea canephora

CcContig1026
MASAFPHLLLLTTLAAICLFSDVPSAALGGRPKDALVGGWSKADPKDPEVLENGKFAID
EHNKEAGTKLEFKTVVEAQEQVVAGTNYKIVIKALDGTASNLYEAIVWVKPWLKFKKLT
SFRKL

CcContig6451
MTEVTVNYNFNITEVAANMAVEGFQSVEAEAIMKTAGDDMIWNAIEDTKDMDMCDEDFF
VTGGGKDTKLVGIAGVPLPKPVDKTSPHVIKIAQFAVKKHNEKAGTKLVFIKVVGGVKW
SAIAGTFYALQIETQDSKGTHRDKTLVVEAITGHKKLIWYKH

CcContig7844
MAAVVANPHINISEITANMKAEGVQSPEIEAIVKALSDDTIWNTIEGFKGKDMSTQEKM
INNMVAGGHLPQVGVPLPTPVNPTDPHVISVAKFAVAKYNDKHGTKLVFNRVNGGLQWK
IVIGTLYILILATQDSKGTYTDYAVVFETFLGQKYLFWYK
