# Supplementary material for: An EST-based analysis identifies new genes and reveals distinctive gene expression features of Coffea arabica and Coffea canephora
Source: BMC Plant Biol. 2011 Feb 8;11:30. doi: 10.1186/1471-2229-11-30 (PMC3045888; doi:10.1186/1471-2229-11-30)
Supplement: Additional file 13 — OrthoMCL families of PinII Serine Proteinase Inhibitors. Word file containing the sequences of PinII Serine Proteinase Inhibitors in coffee. [file 1471-2229-11-30-S13.PDF]

Additional File 13: *Coffea* spp. OrtoMCL families of PinII serine proteinase inhibitors

Family7241

Coffea arabica

CA00_XX_CL2_115_D10_JF_F
MAINKIGAMAILFCGMILLGANVEVTAVRPGPEQICPLYCIVGIEYVDCDGEKTYTDCT
NCCFENGCTLHFKDGTSYFCTWPAKQELGFGKGVYKI

CaContig12344
MAINKIGAMAILFCGMILLGANVEVKAVRPGPGPVCPQYCILGIEYVDCDGEKIYTDCT
NCCLSEGCTLHFTDGTEEYCEPVGKGVYKI

CaContig5418
MMAVNKIGAMAILFCGMILLGANVEVTAVRPGPDQICPLYCIVGIEYIVCDGEKIYTDC
TNCCFANGCTLHFTDGTSYYCTWPAQQELGYGKGVYKI

CaContig13131
MAINKIGAMAILFCGMILLGANVEVTAVRPGPEQICPLYCIVGIEYVDCDGEKTYTDCT
NCCFENGCTLHFKDGTSYFCTWPAKQELGFGKGVYKI

CaContig7989
MAVNKIGAMAILFCGMILLGANVEVTAVRPVPQICPLYCILGIEYVVCDGEKTYKGCTN
CCFENGCTLHFEDGTEKYCTWPTEQKLGLANIMLNNMPF

Coffea canephora

CC00_XX_PP1_063_C07_TL_F
MAVNKIGAMVILFCGMILLGANVEVTAVRPGPEQICPLYCIVGIEYVDCDGEKTYTDCT
NCCFENGCTLHFKDGTSYFCTWPAKHELGFGKGVYKI

Family10273

Coffea arabica

CA00_XX_CA1_003_B05_EZ_F
MAINKIGAMAILFCGMILLGANVEVKAVRPGPVRPCPRNCIGGTLYQICNGTKTYTTCT
NCCVSDGCTLYFLDGSSLYCDWPDAKY

CaContig6030
MGINKIGAMAILFCGMILLGANVEVKAVRPGLLQPCPRNCIGGTVFQICNGTKTYTTCT
NCCVSNGCTLYFLDGSSLYCDWPDAKY

CaContig2158
MAINKIGAMAILFCGMILLGANIEVKAVRQAPLRPCPRNCIGGTVYKVCNGTKTYTDCT
NCCVSDGCTLYFEDGSSLYCDWPYAKY

CaContig14018
MILLSSNVEVKVVEACPQYCLDVEYMTCGNSETKLPPRCNCCLAPKGCTLHLADGTSQY
CS

Coffea canephora

CcContig3974
MAISKIGAMAILFCGMILLGANVEVMAVRPGPIRPCPLICLLTEYKICNGTKTYTNCTN
CCVDDGCTLYFEDGSSIYCEWPWAKY
